# Synthesis and Inhibition Activity Study of Triazinyl-Substituted Amino(alkyl)-benzenesulfonamide Conjugates with Polar and Hydrophobic Amino Acids as Inhibitors of Human Carbonic Anhydrases I, II, IV, IX, and XII

**DOI:** 10.3390/ijms222011283

**Published:** 2021-10-19

**Authors:** Mária Bodnár Mikulová, Dáša Kružlicová, Daniel Pecher, Andrea Petreni, Claudiu T. Supuran, Peter Mikuš

**Affiliations:** 1 Department of Pharmaceutical Analysis and Nuclear Pharmacy, Faculty of Pharmacy, Comenius University in Bratislava, Odbojárov 10, 832 32 Bratislava, Slovakia; mikulova43@uniba.sk (M.B.M.); kruzlicova@fpharm.uniba.sk (D.K.); pecher1@uniba.sk (D.P.); 2 Toxicological and Antidoping Center, Faculty of Pharmacy, Comenius University in Bratislava, Odbojárov 10, 832 32 Bratislava, Slovakia; 3 Neurofarba Department, Section of Pharmaceutical and Nutriceutical Sciences, University of Florence, 50139 Florence, Italy; andrea.petreni@unifi.it (A.P.); claudiu.supuran@unifi.it (C.T.S.)

**Keywords:** 1,3,5-triazine, amino acids, benzenesulfonamide, human carbonic anhydrase, inhibition, hybrid molecule

## Abstract

Primary sulfonamide derivatives with various heterocycles represent the most widespread group of potential human carbonic anhydrase (hCA) inhibitors with high affinity and selectivity towards specific isozymes from the hCA family. In this work, new 4-aminomethyl- and aminoethyl-benzenesulfonamide derivatives with 1,3,5-triazine disubstituted with a pair of identical amino acids, possessing a polar (Ser, Thr, Asn, Gln) and non-polar (Ala, Tyr, Trp) side chain, have been synthesized. The optimized synthetic, purification, and isolation procedures provided several pronounced benefits such as a short reaction time (in sodium bicarbonate aqueous medium), satisfactory yields for the majority of new products (20.6–91.8%, average 60.4%), an effective, well defined semi-preparative RP-C18 liquid chromatography (LC) isolation of desired products with a high purity (>97%), as well as preservation of green chemistry principles. These newly synthesized conjugates, plus their 4-aminobenzenesulfonamide analogues prepared previously, have been investigated in in vitro inhibition studies towards hCA I, II, IV and tumor-associated isozymes IX and XII. The experimental results revealed the strongest inhibition of hCA XII with low nanomolar inhibitory constants (K_i_s) for the derivatives with amino acids possessing non-polar side chains (7.5–9.6 nM). Various derivatives were also promising for some other isozymes.

## 1. Introduction

The incidence of oncological diseases has been constantly growing over recent years, while they have been ranked among the leading causes of death globally. The hypoxic state, with hCA IX and hCA XII as its significant markers, is a common negative attribute of cancer [1]. The family of hCA isozymes catalyze a reversible reaction between carbon dioxide and hydrogen and bicarbonate ions [2]. hCA IX plays a substantial role in pH regulation, adhesion, proliferation, migration, and invasion of tumor cells and correlates with poor patient prognosis and onco-therapy resistance [3,4]. There are many other well-known tumor-specific receptors and markers such as somatostatin, bombesin, glucagon-like peptide 1, prostate-specific membrane antigen (PSMA) and fibroblast activation protein (FAP) [5]. The structural modification and development of novel small-molecule- or monoclonal-antibody-based compounds, with affinity and selectivity towards tumor-specific receptors/markers, belong to the most prospective approaches in the diagnosis, imaging, and therapy of hypoxic tumors, and so for personalized care of oncological patients [5]. Thus, a mechanism of the catalytic activity of tumor-associated hCA isozymes in hypoxic tumors and its inhibition has been a promising target over a long period in many studies by Supuran [6,7,8,9], Pastorek and Pastorekova [10,11,12,13] working groups and in other very recently published papers [14,15,16,17,18]. Anyway, considering a broader extent of disease (also involving non-oncological ones), the inhibition of other hCA isozymes is also useful, e.g., hCA I is related to retinal and cerebral edema, hCA II to glaucoma, bone and renal diseases, edema, epilepsy, or acute high-altitude illness, and hCA IV to glaucoma, stroke, and retinitis [19].

Nowadays, a great effort is still being made in structural modification and design of benzenesulfonamide hCA inhibitors (hCAi), with potential antitumor effects to improve physicochemical and pharmacokinetic properties. The newest studies employed thiazolone-benzenesulfonamides showing the best K_i_ (hCA IX) of 10.93–25.06 nM for three derivatives [14], K_i_ of 7 nM for carbohydrate-based benzenesulfonamide [17], a K_i_ of 19.6 nM for a pyrrolobenzenesulfonamide derivative [18], phenylureidobenzenesulfonamides as analogues of clinically tested SLC-0111 with K_i_s (IX and XII) of 2.59/7.64 nM [16], or quinazoline benzenesulfonamides with top K_i_s (IX and XII) of 13.0–40.7/8.0–10.8 nM [20]. The conjugation of 1,3,5-triazine moiety with benzenesulfonamide has been more frequently explored in recent years to provide effective hCAi. Lolak et al. prepared benzenesulfonamides incorporating 1,3,5-triazine substituted with anilines, or piperidine [21], which effectively inhibited hCA I and II with K_i_s of 51.67 ± 4.76 and 40.35 ± 5.74 nM, while ureidosulfonamides [22,23] inhibited hCA IX with K_i_s of 0.91–126.2 nM. Havránková et al. [24,25,26] developed substituted s-triazines, containing sulfanilamide, homosulfanilamide, 4-aminoethylbenzenesulfonamide, piperazines as well as aminoalcohol moieties, inhibiting hCA IX with K_i_s in the range of 0.4–307.7 nM. Our working group, around Mikuš et al., has brought the first systematic study of 1,3,5-triazinyl-substituted benzenesulfonamides with amino acids as the substituents [27,28,29]. Here, the conjugates possessing a symmetric pair of Gly, β-Ala, Val, Leu, Ile, Met, Pro, Phe, Asp, Glu, showed K_i_s (hCA IX) in the range of 8.4–2592 nM and a significant selectivity towards hCA IX for the derivative with Asp.

An aim of the present work was to prepare and study a new series of 4-aminomethyl- and aminoethyl-derivatives of benzenesulfonamide with 1,3,5-triazine incorporating a pair of identical amino acids with polar (Ser, Thr, Asn, Gln) and non-polar (Ala, Tyr, Trp) side chains. The synthetic strategies using water- as well as organic-solvent-based reaction conditions were investigated. New semi-preparative liquid chromatography (LC) methods with a reversed phase system (RP-C18) or hydrophilic interaction system (HILIC) were developed for highly effective isolation and purification of the desired products. The new sulfonamide derivatives along with their 4-aminobenzenesulfonamide analogues (prepared previously) were evaluated by in vitro inhibition experiments, testing their activity against five hCA isozymes (hCA I, II, IV, IX, and XII). A molecular modelling study was carried out for the visualization of tentative structures of new derivative and interactions in selected associates.

## 2. Results

### 2.1. Chemistry

**Scheme 1:**
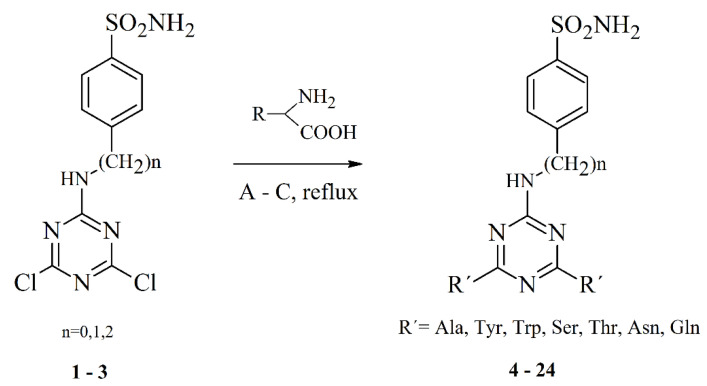
Synthesis of 1,3,5-triazinyl-substituted benzenesulfonamide derivatives (**4**–**24**) using various reaction conditions: (A) Na_2_CO_3_ in water; (B) NaHCO_3_ in water; (C) 1,4-dioxane/TEA.

New derivatives of 4-[((4′,6′-dichloro-1′,3′,5′-triazine-2′-yl)amino)methyl/-2-amino)ethyl]benzenesulfonamide possessing a pair of identical amino acid with a non-polar (Ala, Tyr, Trp) and a polar side chain (Gln, Asn, Ser, Thr) were synthesized using a two-step nucleophilic substitution of chlorine atoms of cyanuric chloride. Initially, starting precursors **1**–**3** were prepared, using well-defined syntheses with acetone and sodium hydroxide at 0 °C, in compliance with the procedure described by Carta et al. [30]. In the second step, several reaction conditions for the substitution of two chlorines of precursors such as type of reaction environment, type of basic catalyst, reactant amounts of substance, or a reaction time, were tested to obtain high yields and purity of desired products (see Scheme 1).

The final optimized conditions primarily depended on the polarity of the amino acid substituent, as summarized in Table 1 at the end of this section. The 4-aminobenzenesulfonamide derivatives with Ala **4**, Trp **6**, Ser **7**, Thr **8** were obtained in high yields (73–99%) after synthesis with satisfactory purity of crude products (87–96.8%), while derivatives with Tyr **5**, Asn **9** and Gln **10** had satisfactory yields (77–96.7%) but lower purity of crude products (21.6–49.7%), for more detailed results see our previous study [29]. Analogically, the previously developed synthetic methods A–C were adapted in this work for synthesis of new 4-aminomethyl- (**11**–**17**) and 4-aminoethyl- (**18**–**24**) benzenesulfonamide derivatives. The yields and purity of crude products were in good agreement with those of **4**–**10**, for the derivatives with aminomethyl linker between 1,3,5-triazine and benzenesulfonamide 88.5–99.8%/25.6–90.7% and for derivatives with aminoethyl linker 68–99.5%/22–92.6%. A water reaction environment with sodium carbonate was included as the optimal synthetic procedure for the derivatives with amino acids with a non-polar side chain, such as Ala, Trp, then with sodium bicarbonate for the derivatives of amino acids with a polar side chain such as Ser, Thr, Asn, Gln, while 1,4-dioxane with triethylamine was used for the tyrosine derivatives (see summary in Table 1). Synthetic procedures with environmentally friendly solvents, such as water, form a main principle of green chemistry. Here, 1,3,5-triazinyl-amino/methyl/ethyl/benzenesulfonamide conjugates disubstituted with a symmetric pair of amino acids (Ala, Trp, Ser, Thr, Asn, Gln) can be advantageously prepared in water environment containing sodium carbonate or sodium bicarbonate (depending on the type of implemented amino acid). For these derivatives, the water-based reactions provided pronounced benefits in terms of higher product yields and purities, shorter reaction times and environmentally friendly (green) synthetic conditions compared to the organic media.

After the synthetic step, proper isolation and purification semi-preparative LC methods for each individual derivative were developed. Various separation parameters, such as selection of stationary phase (RP-C18/HILIC), mobile phase composition (i.e., concentration ratio of water mobile phase A and organic mobile phase B), flow rate, as well as injected volume of a sample, were carefully optimized to avoid any co-elution of structurally related compounds present in the sample (for the detected impurities see Table 1) and to obtain a highly pure desired product. The most effective separation and isolation of desired products were achieved by the isocratic elution on a reverse-phase system (RP-C18) with ammonium bicarbonate as MP A (c = 50 or 100 mmol/L) and methanol or acetonitrile as MP B (4–40%), for the employed procedures and optimized semi-preparative LC methods see Section 3.1. The high separation efficiency of the developed LC-DAD methods was a key factor for the isolation of desired compounds from a mixture with a higher portion of structurally related compounds. The developed purification procedures for triazine derivatives represent an advanced alternative to conventional separation processes, such as crystallization, in terms of well-defined separation, significant increases of purity and lower yield losses as well as collection and reuse of a mobile phase, which meets the requirements of green chemistry. The purified products were subsequently lyophilized to obtain the desired compounds with a purity of 97% (see data in Table 1). The structures and purities of desired products were confirmed by ^1^H- and ^13^C-NMR, HPLC-DAD/MS and IR spectral analyses (see Section 3.2.2). The hCA inhibitory activity was subsequently evaluated for each highly pure derivative (see Section 2.2). 

### 2.2. hCA Inhibition Studies

Inhibition data against cytosolic hCA isozyme I and II, transmembrane isozyme IV, and tumor-associated transmembrane isozymes IX and XII in the presence of new 4-amino(alkyl) derivatives of benzenesulfonamide with 1,3,5-triazine disubstituted with a pair of identical amino acids, possessing polar (Ser, Thr, Asn, Gln) and non-polar (Ala, Tyr, Trp) side chains are evaluated and shown in Table 2. Their 4-aminobenzenesulfonamide analogues which were prepared previously, but not experimentally analyzed for their hCA inhibition activity, are also presented in Table 2. hCA inhibition studies were performed using stopped-flow, CO_2_ hydrase assays [31].

The following results from hCA inhibition studies can be observed:

(1) tested products were only weak inhibitors of hCA I (K_i_ ≥ 340 nM), except for derivatives with Tyr **5**, **12**, **19** and Trp **6**, **13**, **20** (30–98 nM);

(2) very similar data are shown in the inhibition of physiologically relevant hCA II, where only derivatives with Trp **6**, **13**, **20** strongly inhibited this isozyme (1–6 nM);

(3) hCA IV, associated with eye diseases, was resistant against the derivatives with amino acids Ser, Thr, Asn, Gln (K_i_ over 4100 nM), while the derivatives with amino linker and Tyr **5** and Trp **6** inhibited hCA IV at moderate levels (59.3 and 74.6 nM respectively);

(4) miscellaneous results are shown in hCA IX inhibition, where the derivatives with amino acids possessing non-polar side chains inhibited hCA IX at moderate levels (45–95.3 nM), but other derivatives at weak levels (over 260 nM);

(5) the tested compounds showed impressive inhibition of hCA XII, with K_i_s in the range of 7.5–9.4 nM for the derivatives possessing a non-polar side chain (Ala, Tyr and Trp), and 27.1–81.5 nM for the derivatives possessing a polar side chain (Ser, Thr, Asn and Gln).

Consideration of the structure–inhibition activity relationship of the studied derivatives implies that: (i) prolongation of the linker between 1,3,5-triazine and benzenesulfonamide does not have a significant impact on strong inhibition of hCA XII; (ii) introduction of non-polar side chain on amino acid residues enhanced the inhibitory activity against hCA XII in comparison to polar side chains; (iii) in case of hCA I, II, IV, and partially hCA IX, the inhibition effect within the group with less polar derivatives significantly increased with the bulk of amino acid side chain, while this factor did not play any role in the inhibition activity against hCA XII.

It can be summarized that the studied 1,3,5-triazines with 4-amino/methyl/ethyl-benzenesulfonamide and a pair of identical amino acids are able to inhibit important hCA isozymes at a very high to moderate level. Nevertheless, selectivity towards tumor-associated isozymes remains challenging. The derivatives with Trp provided the most consistent inhibition data, acting as potential dominant inhibitors of hCA II and XII. In comparison, clinically tested ureidobenzenesulfonamide demonstrated in an in vitro inhibition study K_i_ (hCA IX) of 45.1 nM and selectivity (II/IX) of 21.3 [32,33]. Within the 1,3,5-triazine benzensulfonamides, etoxy-substituents with amino linker (120 pM) [34] and 4-aminophenol with aminomethyl linker (400 pM) [24] exhibited a promising in vitro picomolar results towards hCA IX with the selectivities of 166.7 and 18.5, respectively. Anyways, when comparing our results with the majority of studies providing K_i_s in the hundreds of nM to tens of mM, the group of 1,3,5-triazines with 4-amino/methyl/ethyl-benzenesulfonamide and amino acids substituents represents a promising structural motif for hCA inhibition worthy of further investigation.

### 2.3. Molecular Modelling

The structure of 1,3,5-triazinyl-aminobenzenesulfonamide derivatives disubstituted with two Trp were visualized and docked into the human carbonic anhydrase isozymes hCA II, hCA IX, and hCA XII. The Trp derivative (ligand) was selected as one of the most potent inhibitors of these important hCA isozymes within the studied group of the derivatives. To begin with, the molecular structure of the chosen sulfonamide ligand was drawn using ChemSketch software [35], which was subsequently exported to Spartan ’14 [36] in order to build up and optimize the 3D structure. During the optimization process, the semi-empirical quantum method PM3 was utilized for finding the final conformer with the lowest energy. The effects of the solvent as well as protonation were taken into account during the modelling process. The best conformer was saved as a mol2 file for later use in the docking software Maestro [37].

Pre-prepared ligands were further processed in the LigPrep package [38] with respect to their geometry (geometry optimization by OPLS3, generation of all optical isomers, low-energy ring conformations), and protonation states (according to residue pKas), and the most convenient structure was selected for final docking into the particular human carbonic anhydrase isozyme (hCA II, hCA IX, or hCA XII). Preparation of carbonic anhydrase proteins involved the import of macromolecular structures from the Protein Data Bank site as well as pre-treatment of imported chains by the Protein Preparation Wizard [39] package (using the 2.2 Å resolution). For later docking, the structure with the PDB entry 3K34 was used as a basis for hCA II, PDB entry 5FL4 for hCA IX, and PDB entry 4HT2 for hCA XII build up. Pre-processing of chains typically involved removing water, hets, fixing of missing chains, etc. The ligand and the protein thus prepared were used for subsequent docking. The sulfonamide group was N-deprotonated, as it is well known that in this form it serves as a zinc binding site. The complex of docked ligand onto protein was minimized by MacroModel [40] using the OPLS3e force field, where constraints were applied to the backbone as well as to the side chains of the protein.

**Figure 1:**
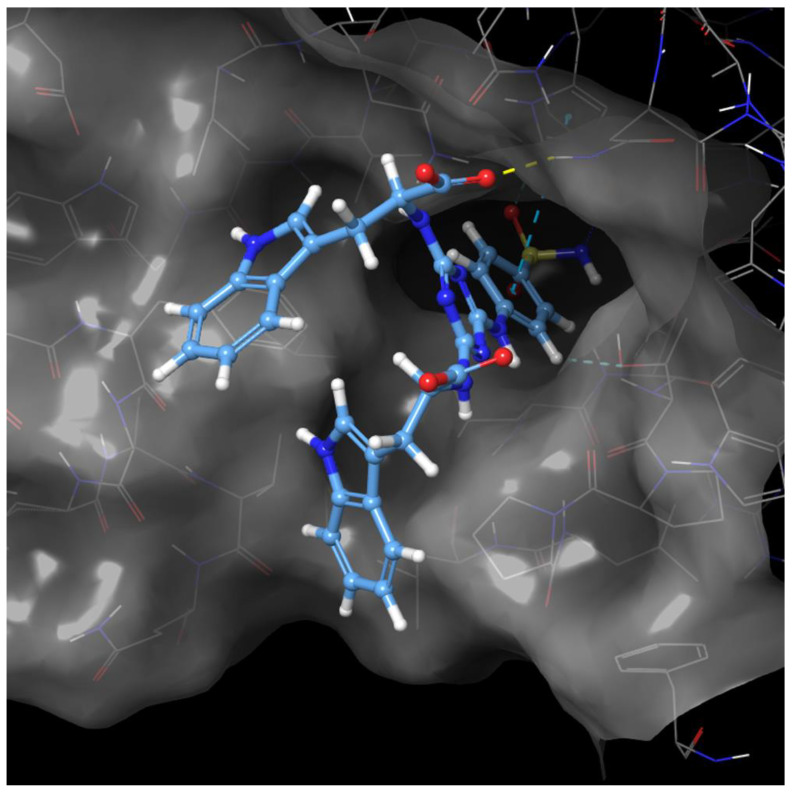
Molecular surface of the binding site of hCA II with docked compound **6** (Trp derivative).

**Figure 2:**
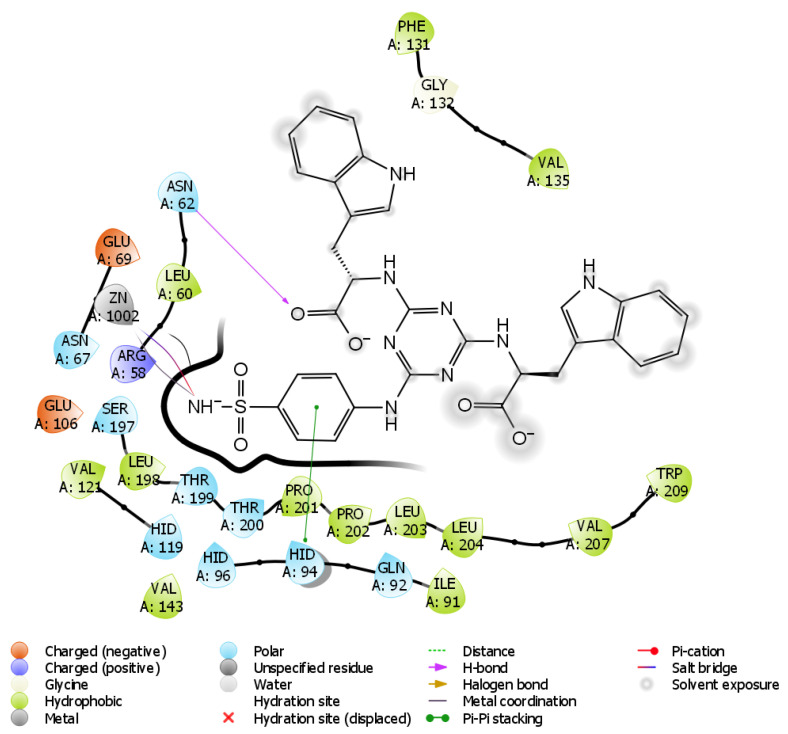
Intermolecular interactions of compound **6** (Trp derivative) docked into hCA II.

**Figure 3:**
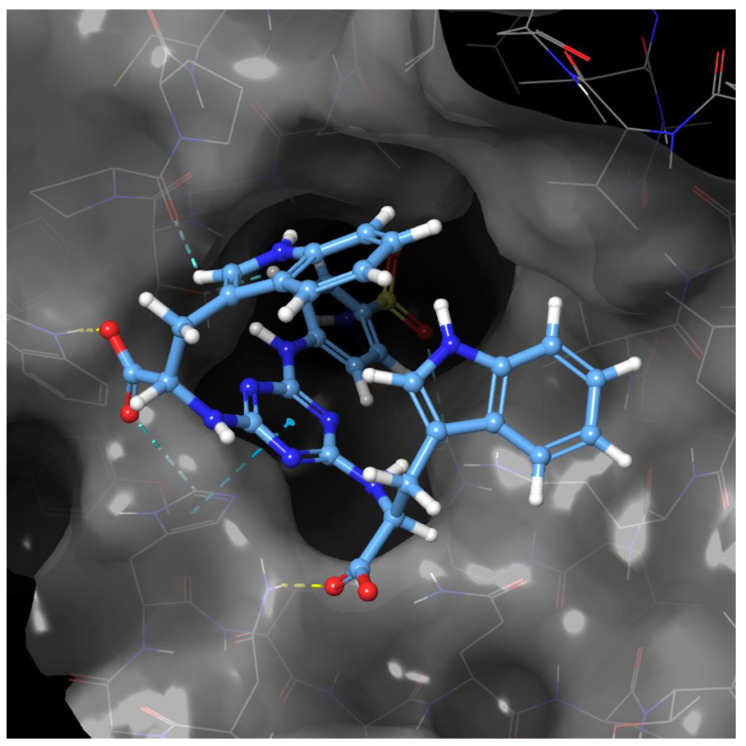
Molecular surface of the binding site of hCA IX with docked compound **6** (Trp derivative).

**Figure 4:**
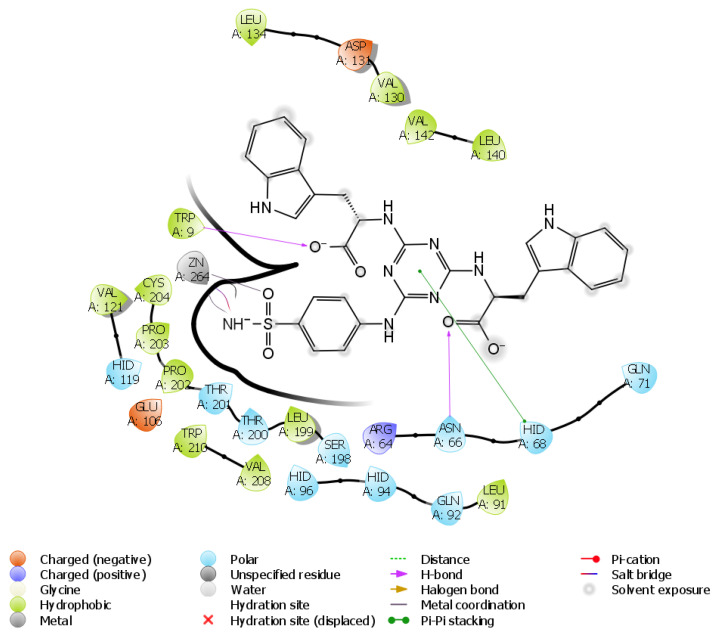
Intermolecular interactions of compound **6** (Trp derivative) docked into hCA IX.

**Figure 5:**
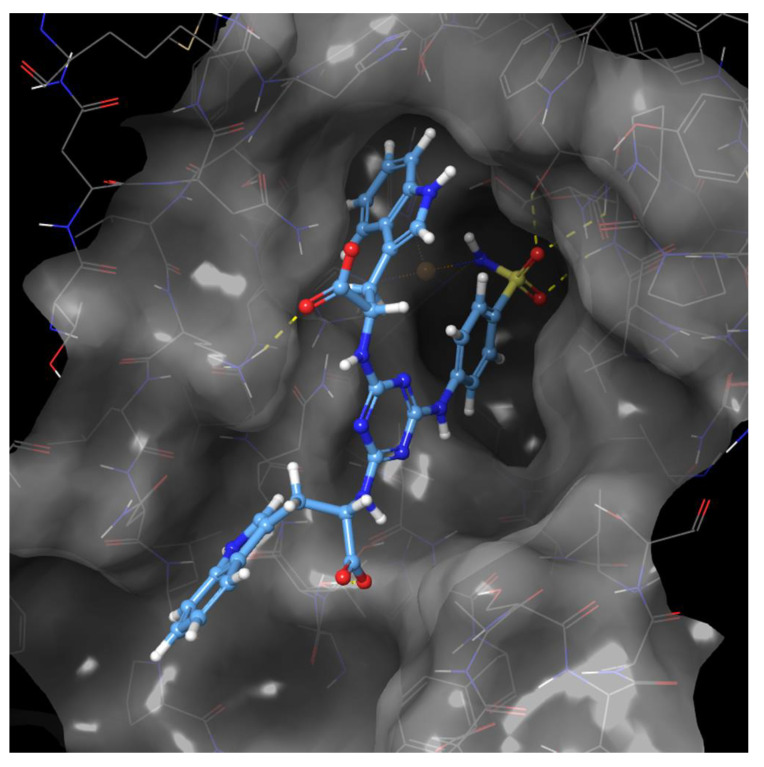
Molecular surface of the binding site of hCA XII with docked compound **6** (Trp derivative).

**Figure 6:**
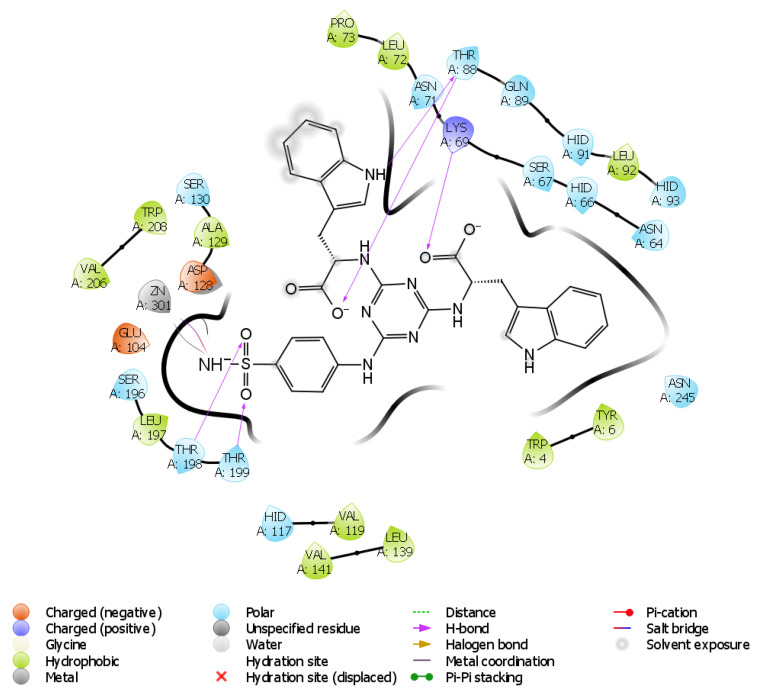
Intermolecular interactions of compound **6** (Trp derivative) docked into hCA XII.

A 3D depiction of the molecular surface of the Trp derivative docked onto the hCA II binding site is shown in Figure 1. Intermolecular interactions of the Trp derivative docked into human isoenzyme II are shown in Figure 2. The aromatic cycle forms a π-π interaction with the histidine 94. The asparagine 62 is attached to the carboxylic group of the Trp residue of the ligand. The orientation of the ligand onto the hCA IX binding site is slightly different from that of the hCA II (Figure 3 and Figure 4). The histidine 68 forms a π-π interaction with the triazine cycle. One hydrogen bond is created between asparagine 66 and the carboxylic group, another H-bond can be observed between tryptophan 9 and the carboxylic group of the ligand. The pose of the ligand docked onto hCA XII is distinct from the poses of hCA II and hCA IX (Figure 5 and Figure 6). Two proton-donor and proton-acceptor H-bonds are created between the sulfonamide functional group and threonines 198 and 199. The first carboxylic group of the Trp residue in the ligand creates a hydrogen bond with lysine 69. The second carboxylic group of the ligand forms one H-bond with threonine 88. The threonine 88 is also responsible for another H-bond with the amino group of indole in the Trp residue.

The binding free energies (ΔG_bind_) were calculated with the use of molecular mechanics/generalized Born surface area (MM-GBSA) energy properties in Prime package [41]. These properties are split down into contributions from several variables in the energy expression and report energies for the ligand, receptor, and complex structures, as well as energy differences relating to strain and binding. The binding free energies for three different proteins, hCA II, hCA IX, and hCA XII, with benzenesulfonamide derivative disubstituted with Trp were obtained as a parameter MMGBSA dG Bind(NS) [kJ/mol] which was calculated as the energy of the optimized complex minus both energies of the receptor and ligand (both calculated from optimized complex). Calculated values for all three hCA complexes were as follows: ΔG_bind_ (hCA II) = −219.74 kJ/mol, ΔG_bind_ (hCA IX) = −156.70 kJ/mol, ΔG_bind_ (hCA XII) = −225.14 kJ/mol. The complexes with lower free binding energies (i.e., Trp-hCA II and Trp-hCA XII) also exhibited lower experimentally obtained inhibition constants (1.61 and 8.21 nM, respectively), while the complex with a higher free binding energy (i.e., Trp-hCA IX) exhibited a higher inhibition constant (45.2 nM).

## 3. Materials and Methods 

### 3.1. Materials and Instruments in the Synthetic, Purification and Analysis Methods

All solvents and reagents were purchased from commercial suppliers (AppliChem GmbH, Darmstadt, Germany; Sigma Aldrich, St. Louis, MO, USA; and VWR International, Vienna, Austria) and used without further purification. 

All synthetic reactions were monitored using thin-layer chromatography (TLC) on the Silica gel plates 60 F254 (Merck, Darmstadt, Germany) with the UV visualization (254 nm) and methanol/chloroform (3:1, *v*/*v*) as eluent.

The synthesized crude products were purified by a semi-preparative LC on a Shimadzu instrument containing two quaternary Prep-LC pumps LC-20AP, autosampler for preparative injection SIL-10AP, column oven CTO-20A, PDA detector SPD-M20A, and fraction collector FRC-10A (Shimadzu, Kyoto, Japan). The semi-preparative LC was performed employing either a RP-C18 column (Kromasil C18, 10 µm, 250 × 10mm, Nouryon, Bohus, Sweden) or a HILIC column (Kromasil 60-10 Sil, 10 µm, 250 × 10mm, Nouryon, Bohus, Sweden) with a mixture of 50 mM/100mM solution of ammonium bicarbonate (mobile phase A, NH_4_HCO_3_) and methanol/acetonitrile (mobile phase B) in isocratic conditions optimized individually for each 4-aminomethyl-/ethyl- benzenesulfonamide derivative (**11**–**24**). For further details, see Table 3. The chromatographically prepared fractions, containing a desired product, were pre-frozen and lyophilized for the required time with the collector temperature set at −84 °C and pressure 0.01 mbar (FreeZone 2.5 Liter Benchtop system, Labconco Corporation, Kansas City, MO, USA) to obtain the desired solid products with high purity of >97% (for purity data see Section 3.2.2. Pure Products Characterization). For purification conditions of the first series of 4-aminobenzenesulfoanmide derivatives **4**–**10**, see our previously reported paper [29].

The nuclear magnetic resonance (^1^H NMR, ^13^C NMR) spectra were measured on an Agilent MR400 (400 MHz) spectrometer (Agilent Technologies, Santa Clara, CA, USA). Samples were dissolved in DMSO-*d*_6_. All NMR analyses were referenced to tetramethylsilane (TMS, δ = 0.00 ppm) as an internal standard and performed at laboratory temperature or 90 °C. The multiplicity is reported as follows: b = broad, s = singlet, d = doublet, t = triplet, q = quartet, dd = doublet of doublet, m = multiplet. 

Infrared (IR) spectra (with KBr optics) were recorded on a Spectrum UATR Two FT-IR spectrometer (PerkinElmer Ltd., Beaconsfield, UK) in the range of 450–4000 cm^−1^ using the Spectrum 10^TM^ Software. Samples in dry solid form were placed on a crystal directly. The intensity of absorption bands is expressed as follows: br—broad, w—weak, m—medium, s—strong. 

All of the LC-DAD/MS data were obtained on an LC Agilent Infinity System (Agilent Technologies, Santa Clara, CA, USA) equipped with a gradient pump (1290 Bin Pump), an automatic injector (1260 HiPals), and a column thermostat (1290 TCC). The LC system was coupled with a photodiode array detector (Infinity 1290 DAD) and a quadrupole time-of-flight mass spectrometer (6520 Accurate Mass Q-TOF LC/MS). Q-TOF was equipped with an electrospray ionization source operated in positive ionization mode. All measurements were performed with the following MS parameters: drying gas temperature 360 °C, drying gas flow 12 L/min, nebulizing gas pressure 60 psi, ESI source voltage 3500 V, fragmentor voltage 100 V, collision gas N_2_. The mass spectrometer was tuned using external calibration before the analysis. For the purity evaluation of the compounds, the peak areas from HPLC-DAD analysis at the wavelength of 254 nm were used.

The HPLC analyses were performed using SeQuant^®^ ZIC^®^-HILIC column, 2.1 × 100 mm, 3.5 μm (Merck KGaA, Darmstadt, Germany) as a stationary phase and a 10 mM ammonium acetate aqueous solution with an addition of 0.1% acetic acid (*v*/*v*) (A) and 100% acetonitrile (B) as mobile phases. The elution was performed in a gradient mode using the following composition of the mobile phases: 0.0 min–95% B; 0.0–20.0 min–linear change to 40% B; 20.0–25.0 min–40% B. The column was re-equilibrated with the initial composition of the mobile phases for 6 min. The flow rate was 0.500 mL/min, and the column was kept at a temperature of 40 °C.

For each sample, 1.0 mg of the solid product was dissolved in 1.00 mL of 80% acetonitrile solution with an addition of 5 mM ammonium bicarbonate. The dissolved samples were filtered through a 0.22 μm nylon syringe filter and a 1.0 μL volume was used for the HPLC-DAD/MS analysis. 

### 3.2. Synthetic Procedures and Pure Products Characterization

The initial precursors **1**–**3** were routinely synthesized according to well-defined procedures in [30]. Synthetic strategies for the first series of 4-aminobenzenesulfonamide derivatives **4**–**10** were published in our previous paper [29] using a water-based as well as organic-solvent-based reaction environment. Subsequently, the optimized synthetic procedures for the preparation of compounds **11**–**24** were applied (see description of methods A, B, C in Section 3.2.1; the product characterization in Section 3.2.2; and the HPLC-DAD/MS, NMR, and IR spectra of the products 11–24 in the Supplementary Material, Sections S1–S14). 

#### 3.2.1. Methods for the Synthesis of 4-[((4′,6′-Dichloro-1′,3′,5′-triazine-2′-yl)amino)methyl/-2-amino)ethyl]benzenesulfonamide Derivatives **11**–**24**

*Method A and B*. A reaction mixture contained the precursor **2**/**3** (1 equiv., 1.5 mmol, 1 mol/L) and amino acid (3 equiv.). In the water-based procedures (sodium carbonate in the method A and bicarbonate in the method B), the reactants were stirred in H_2_O at room temperature for 10 min. The aqueous solution of either Na_2_CO_3_ or NaHCO_3_ (4 equiv.) was added dropwise into the reaction mixture. Then, the mixture was refluxed for 20–24 h (in case of Na_2_CO_3_ procedure) or 6–12 h (in case of NaHCO_3_ procedure) until the completion of reaction was confirmed by TLC. The syntheses were finished by a cooling of the reaction mixture. Acidic hydrolysis by 1M HCl (to proper pH depending on the amino acid) was performed until a maximum amount of the precipitate was produced. The precipitate was filtered off and dried under high vacuum before analysis or purification.

*Method C*. A reaction mixture with the precursor **2**/**3** (1 equiv., 1 mmol, 1 mol/L) in 1,4-dioxane and triethylamine (2.5 equiv.) was stirred for 10 min at room temperature. The amino acid (3 equiv.) was dissolved in a 1,4-dioxane:water solution of sodium carbonate (1:1, *v*/*v*) and added to the stirred reaction mixture. Then, the mixture was refluxed for 12–24 h. The syntheses were finished by cooling the reaction mixture, and the solvent was evaporated under high vacuum. The formed matter was dried under high vacuum before analysis or purification. 

#### 3.2.2. Pure Products Characterization

2,2′-[(6-({4-sulfamoylbenzyl}amino)-1,3,5-triazine-2,4-diyl)diimino]dipropanoic acid **11**

White solid; yield 65.3%. HILIC-DAD purity 98.4%. IR (υ_max_, cm^−1^): 3229 (br w, NH), 2987, 2967 (w, CH), 1720 (w, C=O), 1660 (w), 1630 (w), 1558 (s), 1456 (m), 1405 (m), 1316 (m, SO_2_NH_2_), 1243 (w, C-N), 1159 (s, SO_2_NH_2_). ^1^H NMR (400 MHz, DMSO-*d*_6_, 90 °C) δ ppm: 7.67 (d, 2H, *J* = 8.2 Hz, 2 × Ar-H_a_), 7.38 (d, 2H, *J* = 8.2 Hz, 2 × Ar-H_b_), 6.80 (br s, 1H, **NH**-CH_2_), 6.10 (br s, 2H, 2 × **NH**-CH), 4.40 (s, 2H, NH-**CH_2_**), 4.16 (br s, 2H, 2 × H-C(2)), 1.21 (d, 6H, *J* = 6.3 Hz, 2 × H-C(3)). ^13^C NMR (100.58 MHz, DMSO-*d*_6_, 90 °C) δ ppm: 175.13; 166.32; 165.58; 145.33; 142.85; 127.88; 125.95; 49.84; 43.59; 18.65. HRMS (ESI/QTOF, *m*/*z*): [M + H]^+^ Calcd. for [C_16_H_21_N_7_SO_6_H]^+^ 440.1352; Found: 440.1341.

2,2′-[(6-({4-sulfamoylbenzyl}amino)-1,3,5-triazine-2,4-diyl)diimino]bis(3-(4-hydroxyphenyl)propanoic acid) **12**

Light beige solid; yield 31.3%. HILIC-DAD purity 97.0%. IR (υ_max_, cm^−1^): 3206 (br w, NH), 3023 (br w), 2986 (w, CH), 1718 (w, C=O), 1613 (w), 1557 (s), 1512 (s), 1444 (m), 1395 (m), 1317 (m, SO_2_NH_2_), 1239 (m, C-N), 1154 (s, SO_2_NH_2_). ^1^H-NMR (400 MHz, DMSO-*d*_6_, 90 °C) δ ppm: 7.74 (d, 2H, *J =* 8.3 Hz, 2 × Ar_BSA_-H_a_), 7.43 (d, 2H, *J =* 8.3 Hz, 2 × Ar_BSA_-H_b_), 6.97 (d, 4H, *J* = 8.4 Hz, 4 × Ar_TYR_-H), 6.62 (d, 4H, *J* = 8.4 Hz, 4 × Ar_TYR_-H), 5.97 (br s, 3H, 2 × **NH**-CH, Ar-NH), 4.45 (br s, 4H, 2 × H-C(2), NH-**CH_2_**), 2.97 (dd, 2H, *J* = 14.0 Hz, *J* = 5.4 Hz, 2 × H_a_-C(3)), 2.88 (dd, 2H, *J* = 14.0 Hz, *J* = 7.3 Hz, 2 × H_b_-C(3)). ^13^C NMR (100.58 MHz, DMSO-*d*_6_, 90 °C) δ ppm: 174.09; 166.29; 165.79; 156.22; 145.24; 142.95; 130.43; 128.74; 128.00; 126.04; 115.47; 55.64; 43.66; 36.94. HRMS (ESI/QTOF, *m*/*z)*: [M + H]^+^ Calcd. for [C_28_H_29_N_7_SO_8_H]^+^ 624.1876; Found: 624.1855.

2,2′-[(6-({4-sulfamoylbenzyl}amino)-1,3,5-triazine-2,4-diyl)diimino]bis(3-(1H-indol-3-yl)propanoic acid) **13**

Light beige solid; yield 73.8%. HILIC-DAD purity 97.0%. IR (υ_max_, cm^−1^): 3587 (w), 3264 (br w), 3059 (w), 2932 (w, CH), 1718 (w, C=O), 1622 (m), 1558 (s), 1457 (w), 1403 (m), 1329 (m, SO_2_NH_2_), 1242 (w, C-N), 1156 (s, SO_2_NH_2_). ^1^H NMR (400 MHz, DMSO-*d*_6_, 90 °C) δ ppm: 10.44 (s, 2H, 2 × H-N_ind_), 7.66 (d, 2H, *J* = 8.2 Hz, 2 × Ar-H_a_), 7.42 (d, 2H, *J* = 7.9 Hz, 2 × Ar_ind_-H), 7.35 (d, 2H, *J* = 8.2 Hz, 2 × Ar-H_b_), 7.23 (d, 2H, *J* = 8.1 Hz, 2 × Ar_ind_-H), 7.01 (s, 2H, 2 × Ar_ind_-H), 6.97–6.93 (m, 2H, 2 × Ar_ind_-H), 6.87–6.83 (m, 2H, 2 × Ar_ind_-H), 5.90 (br s, 1H, **NH**-CH_2_), 4.55 (br s, 2H, NH-**CH_2_**), 4.35 (s, 2H, 2 × H-C(2)), 3.16 (dd, 2H, *J* = 14.5 Hz, *J* = 4.8 Hz, 2 × H_a_-C(3)), 3.05 (dd, 2H, *J* = 14.5 Hz, *J* = 6.9 Hz, 2 × H_b_-C(3)). ^13^C-NMR (100.58 MHz, DMSO-*d*_6_, 90 °C) δ ppm: 174.33; 166.23; 165.78; 145.15; 142.86; 136.63; 128.11; 125.97; 123.89; 121.10; 118.64; 111.60; 110.85; 54.80; 43.54; 27.80. HRMS (ESI/QTOF, *m*/*z*): [M + H]^+^ Calcd. for [C_32_H_31_N_9_SO_6_H]^+^ 670.2196; Found: 670.2183.

2,2′-[(6-({4-sulfamoylbenzyl}amino)-1,3,5-triazine-2,4-diyl)diimino]bis(3-hydroxypropanoic acid) **14**

White solid; yield 72.8%. HILIC-DAD purity 98.5%. IR (υ_max_, cm^−1^): 3244 (br w), 3062 (br w), 2967, 2946 (w, CH), 1718 (w, C=O), 1653 (w), 1558 (s), 1516 (m), 1404 (m), 1307 (m, SO_2_NH_2_), 1234 (w, C-N), 1154 (s, SO_2_NH_2_). ^1^H NMR (400 MHz, DMSO-*d*_6_, 90 °C) δ ppm: 7.68 (d, 2H, *J* = 8.3 Hz, 2 × Ar-H_a_), 7.38 (d, 2H, *J* = 8.3 Hz, 2 × Ar-H_b_), 6.91 (br s, 1H, Ar-NH), 5.93 (br s, 2H, 2 × **NH**-CH), 4.40 (s, 2H, NH-**CH_2_**), 4.15–4.12 (m, 2H, 2 × H-C(2)), 3.66 (dd, 2H, *J* = 10.5 Hz, *J* = 5.2 Hz, 2 × H_a_-C(3)), 3.51 (dd, 2H, *J* = 10.5 Hz, *J* = 5.2 Hz, H_b_-C(3)). ^13^C NMR (100.58 MHz, DMSO-*d*_6_, 90 °C) δ ppm: 173.07; 166.37; 165.95; 145.28; 142.97; 128.01; 126.07; 62.95; 56.39; 43.69. HRMS (ESI/QTOF, *m*/*z*): [M + H]^+^ Calcd. for [C_16_H_21_N_7_SO_8_H]^+^ 472.1250; Found: 472.1242.

2,2′-[(6-({4-sulfamoylbenzyl}amino)-1,3,5-triazine-2,4-diyl)diimino]bis(3-hydroxybutanoic acid) **15**

White solid; yield 59.8%. HILIC-DAD purity 98.9%. IR (υ_max_, cm^−1^): 3220 (br w), 2977 (w), 1716 (w, C=O), 1623 (m), 1558 (s), 1508 (m), 1388 (s), 1319 (s, SO_2_NH_2_), 1239 (w, C-N), 1155 (s, SO_2_NH_2_). ^1^H NMR (400 MHz, DMSO-*d*_6_, 90 °C) δ ppm: 7.67 (d, 2H, *J* = 8.3 Hz, 2 × Ar-H_a_), 7.39 (d, 2H, *J* = 8.3 Hz, 2 × Ar-H_b_), 5.72 (br s, 2H, 2 × **NH**-CH), 4.43–4.34 (m, 2H, 2 × H-C(3)), 4.00–3.91 (m, 4H, 2 × H-C(2), NH-**CH_2_**), 0.86 (d, 6H, *J* = 6.1 Hz, 2 × H-C(4)). ^13^C NMR (100.58 MHz, DMSO-*d*_6_, 90 °C) δ ppm: 173.38; 166.53; 165.95; 145.48; 142.91; 128.07; 126.03; 66.79; 58.97; 43.75; 19.50. HRMS (ESI/QTOF, *m*/*z*): [M + H]^+^ Calcd. for [C_18_H_25_N_7_SO_8_H]^+^ 500.1563; Found: 500.1554.

2,2′-[(6-({4-sulfamoylbenzyl}amino)-1,3,5-triazine-2,4-diyl)diimino]bis(3-carbamoylpropanoic acid) **16**

White solid; yield 76.5%. HILIC-DAD purity 98.1%. IR (υ_max_, cm^−1^): 3188 (br w), 3052 (w), 1655 (m), 1558 (s), 1510 (m), 1388 (s), 1312 (s, SO_2_NH_2_), 1239 (w, C-N), 1154 (s, SO_2_NH_2_). ^1^H NMR (400 MHz, DMSO-*d*_6_, 90 °C) δ ppm: 7.68 (d, 2H, *J* = 8.3 Hz, 2 × Ar-H_a_), 7.39 (d, 2H, *J* = 8.3 Hz, 2 × Ar-H_b_), 6.86 (br s, 1H, NH), 6.09 (br s, 2H, 2 × **NH**-CH), 4.45–4.36 (m, 4H, 2 × H-C(2), NH-**CH_2_**), 2.47 (d, 4H, *J* = 5.9 Hz, 2 × H-C(3)). ^13^C-NMR (100.58 MHz, DMSO-*d*_6_, 90 °C) δ ppm: 173.84; 172.77; 166.31; 165.68; 145.29; 142.87; 128.05; 126.05; 51.56; 43.66; 38.84. HRMS (ESI/QTOF, *m*/*z*): [M + H]^+^ Calcd. for [C_18_H_23_N_9_SO_8_H]^+^ 526.1468; Found: 526.1449.

2,2′-[(6-({4-sulfamoylbenzyl}amino)-1,3,5-triazine-2,4-diyl)diimino]bis(4-carbamoylbutanoic acid) **17**

White solid; yield 31.6%. HILIC-DAD purity 97.9%. IR (υ_max_, cm^−1^): 3182 (br w), 2968 (w), 2949 (w), 1714 (w, C=O), 1655 (m), 1551 (s), 1508 (s), 1443 (w), 1395 (s), 1311 (s, SO_2_NH_2_), 1241 (w, C-N), 1151 (s, SO_2_NH_2_). ^1^H NMR (400 MHz, DMSO-*d*_6_, 90 °C) δ ppm: 7.75 (d, 2H, *J* = 8.3 Hz, 2 × Ar-H_a_), 7.46 (d, 2H, *J* = 8.3 Hz, 2 × Ar-H_b_), 6.83 (br s, 1H, Ar-NH), 6.09 (br s, 2H, 2 × **NH**-CH), 4.47 (s, 2H, NH-**CH_2_**), 4.17–4.14 (m, 2H, 2 × H-C(2)), 2.20–2.07 (m, 4H, 2 × H-C(4)), 2.01–1.92 (m, 2H, 2 × H_a_-C(3)), 1.90–1.81 (m, 2H, 2 × H_b_-C(3)). ^13^C NMR (100.58 MHz, DMSO-*d*_6_, 90 °C) δ ppm: 174.87; 174.49; 166.36; 165.85; 145.42; 142.87; 128.02; 126.05; 54.43; 43.69; 32.37; 28.66. HRMS (ESI/QTOF, *m*/*z*): [M + H]^+^ Calcd. for [C_20_H_27_N_9_SO_8_H]^+^ 554.1781; Found: 554.1770.

2,2′-[(6-({2-(4-sulfamoylphenyl)ethyl}amino)-1,3,5-triazine-2,4-diyl)diimino]dipropanoic acid **18**

White solid; 82.2%. HILIC-DAD purity 98.5%. IR (υ_max_, cm^−1^): 3280 (br w), 2970 (m), 2937 (w), 1717 (w, C=O), 1628 (w), 1559 (s), 1455 (m), 1405 (m), 1312 (m, SO_2_NH_2_), 1243 (w, C-N), 1158 (s, SO_2_NH_2_). ^1^H NMR (400 MHz, DMSO-*d*_6_, 90 °C) δ ppm: 7.67 (d, 2H, *J* = 8.3 Hz, 2 × Ar-H_a_), 7.32 (d, 2H, *J* = 8.3 Hz, 2 × Ar-H_b_), 6.24 (br s, 1 H, **NH**-CH_2_), 6.12 (br s, 2H, 2 × **NH**-CH), 4.22 (br s, 2H, 2 × H-C(2)), 3.39 (br s, 2H, NH-**CH_2_**), 2.82 (t, 2H, *J* = 7.3 Hz, CH_2_-**CH_2_**-Ar), 1.24 (d, 6H, *J* = 6.5 Hz, 2 × H-C(3)). ^13^C NMR (100.58 MHz, DMSO-*d*_6_, 90 °C) δ ppm: 175.17; 166.17; 165.59; 144.52; 142.46; 129.31; 126.08; 49.64; 41.62; 35.67; 18.49. HRMS (ESI/QTOF, *m*/*z*): [M + H]^+^ Calcd. for [C_17_H_23_N_7_SO_6_H]^+^ 454.1508; Found: 454.1496.

2,2′-[(6-({2-(4-sulfamoylphenyl)ethyl}amino)-1,3,5-triazine-2,4-diyl)diimino]bis(3-(4-hydroxyphenyl)propanoic acid) **19**

Light beige solid; yield 20.6%. HILIC-DAD purity 97.8%. IR (υ_max_, cm^−1^): 3563 (br w), 3232 (br w), 3028 (w), 1716 (w, C=O), 1615 (w), 1557 (s), 1511 (s), 1442 (s), 1393 (m), 1312 (m, SO_2_NH_2_), 1239 (m, C-N), 1154 (s, SO_2_NH_2_). ^1^H-NMR (400 MHz, DMSO-*d*_6_, 90 °C) δ ppm: 7.74 (d, 2H, *J =* 8.3 Hz, 2 × Ar-H_a_), 7.38 (d, 2H, *J =* 8.3 Hz, 2 × Ar-H_b_), 6.99 (d, 4H, *J =* 8.4 Hz, 4 × Ar-H_a_), 6.63 (d, 4H, *J =* 8.4 Hz, 4 × Ar-H_b_), 5.95 (br s, 3H, 2 × **NH**-CH, Ar-NH), 4.50–4.48 (m, 2H, 2 × H-C(2)), 3.46–3.41 (m, 2H, NH-**CH_2_**), 2.99 (dd, 2H, *J* = 13.8 Hz, *J* = 5.4 Hz, 2 × H_a_-C(3)), 2.92–2.85 (m, 4H, 2 × H_b_-C(3), CH_2_-**CH_2_**-Ar). ^13^C-NMR (100.58 MHz, DMSO-*d_6_,* 90 °C) δ ppm: 174.09; 166.14; 165.75; 156.22; 144.54; 142.51; 130.42; 129.35; 128.75; 126.15; 115.45; 55.60; 41.70; 36.94; 35.67. HRMS (ESI/QTOF, *m*/*z*): [M + H]^+^ Calcd. for [C_29_H_31_N_7_SO_8_H]^+^ 638.2033; Found: 638.2010.

2,2′-[(6-({2-(4-sulfamoylphenyl)ethyl}amino)-1,3,5-triazine-2,4-diyl)diimino]bis(3-(1H-indol-3-yl)propanoic acid) **20**

Light beige solid; yield 76.9%. HILIC-DAD purity 98.4%. IR (υ_max_, cm^−1^): 3572 (w), 3241 (br w), 2933 (w), 1718 (w), 1623 (m), 1543 (s), 1467 (w), 1436 (w), 1395 (m), 1311 (m, SO_2_NH_2_), 1232 (w), 1155 (s, SO_2_NH_2_). ^1^H-NMR (400 MHz, DMSO-*d*_6_, 90 °C) δ ppm: 10.42 (s, 2H, 2 × H-N_ind_), 7.65 (d, 2H, *J* = 8.3 Hz, 2 × Ar-H_a_), 7.41 (d, 2H, *J* = 7.9 Hz, 2 × Ar_ind_-H), 7.28 (d, 2H, *J* = 8.3 Hz, 2 × Ar-H_b_), 7.22 (d, 2H, *J* = 7.9 Hz, 2 × Ar_ind_-H), 7.03 (s, 2H, 2 × Ar_ind_-H), 6.96–6.92 (m, 2H, 2 × Ar_ind_-H), 6.86–6.82 (m, 2H, 2 × Ar_ind_-H), 6.20 (br s, 1H, Ar-NH), 5.86 (br s, 2H, **NH**-CH), 4.50 (br s, 2H, 2 × H-C(2)), 3.35–3.31 (m, 2H, NH-**CH_2_**), 3.18 (dd, 2H, *J* = 14.5 Hz, *J* = 5.3 Hz, 2 × H_a_-C(3)), 3.06 (dd, 2H, *J* = 14.5 Hz, *J* = 7.3 Hz, 2 × H_b_-C(3)), 2.77 (t, 2H, *J* = 7.3 Hz, CH_2_-**CH_2_**-Ar). ^13^C-NMR (100.58 MHz, DMSO-*d_6_,* 90 °C) δ ppm: 174.53; 166.22; 165.75; 144.59; 142.49; 136.67; 129.36; 128.37; 126.15; 123.93; 121.05; 118.74; 111.44; 55.21; 41.73; 35.72; 28.00. HRMS (ESI/QTOF, *m*/*z*): [M+H]^+^ Calcd. for [C_33_H_33_N_9_SO_6_H]^+^ 684.2352; Found: 684.2329. 

2,2′-[(6-({2-(4-sulfamoylphenyl)ethyl}amino)-1,3,5-triazine-2,4-diyl)diimino]bis(3-hydroxypropanoic acid) **21**

White solid; yield 72.9%. HILIC-DAD purity 97.5%. IR (υ_max_, cm^−1^): 3226 (br m), 2941 (w), 1728 (w, C=O), 1558 (s), 1515 (w), 1404 (s), 1308 (m, SO_2_NH_2_), 1241 (w, C-N), 1156 (s, SO_2_NH_2_). ^1^H-NMR (400 MHz, DMSO-*d*_6_, 90 °C) δ ppm: 7.67 (d, 2H, *J* = 8.9 Hz, 2 × Ar-H_a_), 7.35 (d, 2H, *J* = 8.9 Hz, 2 × Ar-H_b_), 6.62 (br s, 2H, Ar-NH), 5.99 (br s, 2H, 2 × **NH**-CH), 4.00 (br s, 2H, 2 × H-C(2)), 3.69–3.64 (m, 2H, 2 × H_a_-C(3)), 3.49–3.46 (m, 2H, 2 × H_b_-C(3)), 3.37–3.33 (m, 2H, NH-**CH_2_**), 2.81 (t, 2H, *J* = 7.4 Hz, CH_2_-**CH_2_**-Ar). ^13^C-NMR (100.58 MHz, DMSO-*d_6_,* 90 °C) δ ppm: 173.63; 166.02; 165.64; 144.52; 142.38; 129.49; 126.13; 63.46; 56.30; 41.74; 35.65. HRMS (ESI/QTOF, *m*/*z*): [M+H]^+^ Calcd. for [C_17_H_23_N_7_SO_8_H]^+^ 486.1407; Found: 486.1391.

2,2′-[(6-({2-(4-sulfamoylphenyl)ethyl}amino)-1,3,5-triazine-2,4-diyl)diimino]bis(3-hydroxybutanoic acid) **22**

White solid; yield 60.5%. HILIC-DAD purity 97.7%. IR (υ_max_, cm^−1^): 3177 (br w), 3047 (br w), 2852 (w), 1715 (w, C=O), 1558 (s), 1496 (m, C=C), 1436 (s), 1356 (br m, SO_2_NH_2_), 1259 (w, C-N), 1156 (s, SO_2_NH_2_). ^1^H-NMR (400 MHz, DMSO-*d*_6_, 90 °C) δ ppm: 7.67 (d, 2H, *J* = 8.2 Hz, 2 × Ar-H_a_), 7.34 (d, 2H, *J* = 8.2 Hz, 2 × Ar-H_b_), 6.19 (br s, 1H, Ar-NH), 5.73 (br s, 2H, 2 × **NH**-CH), 4.02–3.97 (m, 2H, 2 × H-C(3)), 3.92–3.89 (m, 2H, 2 × H-C(2)), 3.42–3.34 (m, 2H, NH-**CH_2_**), 2.82 (t, 2H, *J* = 7.3 Hz, CH_2_-**CH_2_**-Ar), 0.87 (d, 6H, *J* = 6.2 Hz, 2 × H-C(4)). ^13^C-NMR (100.58 MHz, DMSO-*d_6_,* 90 °C) δ ppm: 173.13; 166.34; 164.99; 144.54; 142.56; 129.42; 126.20; 66.99; 59.44; 41.80; 35.72; 20.60. HRMS (ESI/QTOF, *m*/*z*): [M+H]^+^ Calcd. for [C_19_H_27_N_7_SO_8_H]^+^ 514.1720; Found: 514.1707.

2,2′-[(6-({2-(4-sulfamoylphenyl)ethyl}amino)-1,3,5-triazine-2,4-diyl)diimino]bis(3-carbamoylpropanoic acid) **23**

White solid; yield 63.2%. HILIC-DAD purity 98.0%. IR (υ_max_, cm^−1^): 3198 (br m), 3057 (w), 1719 (w), 1660 (w), 1559 (s), 1510 (m, C=C), 1391 (m), 1312 (m, SO_2_NH_2_), 1239 (w, C-N), 1156 (s, SO_2_NH_2_). ^1^H-NMR (400 MHz, DMSO-*d*_6_, 90 °C) δ ppm: 7.74 (d, 2H, *J* = 8.2 Hz, 2 × Ar-H_a_), 7.40 (d, 2H, *J* = 8.2 Hz, 2 × Ar-H_b_), 6.38 (br s, 1H, Ar-NH), 6.17 (br s, 2H, 2 × **NH**-CH), 4.54 (br s, 2H, 2 × H-C(2)), 3.53–3.36 (m, 2H, NH-**CH_2_**), 2.89 (t, 2H, *J* = 7.2 Hz, CH_2_-**CH_2_**-Ar), 2.57 (d, 4H, *J* = 5.9 Hz, 2 × H-C(3)). ^13^C-NMR (100.58 MHz, DMSO-*d_6_,* 90 °C) δ ppm: 173.96; 172.69; 166.21; 165.71; 144.57; 142.49; 129.43; 126.17; 51.42; 41.71; 38.66; 35.75. HRMS (ESI/QTOF, *m*/*z*): [M+H]^+^ Calcd. for [C_19_H_25_N_9_SO_8_H]^+^ 540.1625; Found: 540.1609.

2,2′-[(6-({2-(4-sulfamoylphenyl)ethyl}amino)-1,3,5-triazine-2,4-diyl)diimino]bis(4-carbamoylbutanoic acid) **24**

White solid; yield 29.8%. HILIC-DAD purity 97.1%. IR (υ_max_, cm^−1^): 3195 (br w), 2941 (w), 1723 (w, C=O), 1652 (m), 1557 (s), 1446 (w), 1394 (s), 1310 (s, SO_2_NH_2_), 1243 (w, C-N), 1153 (s, SO_2_NH_2_). ^1^H-NMR (400 MHz, DMSO-*d*_6_, 90 °C) δ ppm: 7.74 (d, 2H, *J* = 8.3 Hz, 2 × Ar-H_a_), 7.40 (d, 2H, *J* = 8.3 Hz, 2 × Ar-H_b_), 6.23 (br s, 1H, Ar-NH), 6.05 (br s, 2H, 2 × **NH**-CH), 4.16 (br s, 2H, 2 × H-C(2)), 3.52–3.45 (m, 2H, NH-**CH_2_**), 2.89 (t, 2H, *J* = 7.3 Hz, CH_2_-**CH_2_**-Ar), 2.21–2.09 (m, 4H, 2 × H-C(4)), 2.04–1.95 (m, 2H, 2 × H_a_-C(3)), 1.92–1.83 (m, 2H, 2 × H_b_-C(3)). ^13^C-NMR (100.58 MHz, DMSO-*d_6_,* 90 °C) δ ppm: 174.96; 171.47; 166.26; 165.78; 144.64; 142.47; 129.40; 126.15; 54.61; 41.71; 35.80; 32.42; 28.85. HRMS (ESI/QTOF, *m*/*z*): [M+H]^+^ Calcd. for [C_21_H_29_N_9_SO_8_H]^+^ 568.1938; Found: 568.1919.

### 3.3. hCA Inhibition Assay

An Applied Photophysics stopped-flow instrument (Applied Photophysics, Leatherhead, UK) was used for assaying the CA-catalyzed CO_2_ hydration activity [31]. Phenol red (at a concentration of 0.2 mM) was used as indicator, working at the absorbance maximum of 557 nm, with 20 mM Tris (pH 8.3) as buffer, and 20 mM Na_2_SO_4_ (for maintaining constant the ionic strength), following the initial rates of the CA-catalyzed CO_2_ hydration reaction for a period of 10–100 s. The CO_2_ concentrations ranged from 1.7 to 17 mM for the determination of the kinetic parameters and inhibition constants. For each inhibitor, at least six traces of the initial 5–10% of the reaction were used for determining the initial velocity. The uncatalyzed rates were determined in the same manner and subtracted from the total observed rates. Stock solutions of inhibitor (0.1 mM) were prepared in distilled-deionized water and dilutions up to 0.005 nM were done thereafter with the assay buffer. Inhibitor and enzyme solutions were preincubated together for 15 min at room temperature prior to assay, in order to allow formation of the E–I complex. The inhibition constants were obtained by non-linear least-squares methods using PRISM 3 and the ChengPrusoff equation, as reported earlier, and represent the mean from at least three different determinations. All CA isozymes were recombinant ones obtained in-house.

### 3.4. Molecular Docking

LigPrep [38] was used to prepare ligand **6** (geometry optimization by OPLS 3, generation of all optical isomers, low-energy ring conformations) for the generation of the probable ionized and tautomerized structures within a pH range of 7 ± 2. The sulfonamide group was N-deprotonated, as it is well known that in this form it serves as a zinc binding site. Maestro program submodule MacroModel [40] was utilized for minimization of docked ligand 6 onto the hCA II, hCA IX, and hCA XII using the OPLS3e force field, with constraints applied to the protein’s backbone as well as its side chains.

## 4. Conclusions

The present work focused on the synthesis, isolation, purification, identification, and hCA inhibition activity study of a series of new 1,3,5-triazinylamino(alkyl)benzenesulfonamides with a pair of identical amino acids possessing non-polar as well as polar side chains. Generally, a water reaction environment with an appropriate base was proved to be a more favorable alternative compared to an organic reaction media in terms of shorter time of synthesis, higher reaction selectivity (reflected in higher yields and purity of desired products), and environmental aspects preserving green chemistry principles. A water reaction environment with sodium carbonate was included as the optimal synthetic condition for derivatives with amino acids with a non-polar side chain, and with sodium bicarbonate for derivatives with amino acids with a polar side chain. The only exceptions were tyrosine derivatives where the organic reaction medium (1,4-dioxane with triethylamine) provided better results than the aqueous one. For the production of derivatives with very high purity, essential for obtaining highly reliable inhibition activity data, a semi-preparative LC method with DAD detection was introduced in this advanced preparation protocol. Thanks to the high separation efficiency of the developed LC-DAD methods, desired products could be effectively isolated from their structurally related analogues (impurities). Moreover, such a well-defined isolation procedure could serve also for the production of some interesting side products of the reaction schemes. High purities (>97%) and chemical structures of the desired products (4–24) were confirmed by HPLC-DAD/MS (ESI-Q-TOF), IR, and ^1^H- and ^13^C-NMR spectral analyses. The obtained inhibition data indicated several promising derivatives with impressive inhibition ability against the tested hCA isozymes, namely I, II, IV, IX, and XII. Here, the derivatives substituted with amino acids possessing non-polar aliphatic as well as aromatic side chains provided the most consistent low nM inhibition data for tumor-associated hCA XII. In case of hCA I, II, IV, and partially hCA IX, the inhibition effect within the group with less polar derivatives significantly increased with the bulk of the amino acid side chain.

In comparison with the majority of the studies providing K_i_s in the hundreds of nM to tens of mM, the group of 1,3,5-triazines with 4-amino/methyl/ethyl-benzenesulfonamide and amino acid substituents represents a promising structural motif for hCA inhibition and, in perspective, will be studied for cytotoxicity and pharmacokinetic properties. A wider range of chemical and biological activities of derivatives (antioxidant, microbial, etc.) could be also expected owing to the main structural units (i.e., triazine, sulfonamide, amino acid), which is worthy of further investigation. Thanks to the natural complexing properties of amino acids, the new triazine derivatives of this group will be tested also for their ability to create metal complexes as precursors of potential (radio)pharmaceuticals.

## Figures and Tables

**Table 1:** Optimized reaction conditions, characteristic impurities of synthesized products, yields, and purities of the LC-purified desired products.

Product (M_r_ in g/mol)	Reaction Conditions	Main Impurities in Crude Products before Purification (HPLC-DAD/MS)	Desired Products after Purification	
			Yield [%]	Purity [%]
**4** T-SA-Ala_2_ (425.42)	A (reflux, 20 h)	OH + AA 1.2%; 438.0669 6.8%	91.8	99.6
**5** T-SA-Tyr_2_ (609.61)	C (reflux, 24 h)	OH + AA 32.2%; OH + OH 2.7%	29.0	98.2
**6** T-SA-Trp_2_ (655.68)	A (reflux, 24 h)	OH + AA 1.8%; Trp 1.6%	87.2	98.4
**7** T-SA-Ser_2_ (457.42)	B (reflux, 7 h)	OH + AA 2.3%	71.0	98.1
**8** T-SA-Thr_2_ (485.47)	B (reflux, 7 h)	OH + AA 1.4%	87.2	99.3
**9** T-SA-Asn_2_ (511.47)	B (reflux, 12 h)	OH + AA 0.4%; OH + OH 1.3%	56.0	98.1
**10** T-SA-Gln_2_ (539.52)	B (reflux, 12 h)	OH + AA 9.4%; AA + Cl 4.9%	28.4	98.0
**11** T-MSA-Ala_2_ (439.45)	A (reflux, 24 h)	OH + AA 1.1%; OH + OH 0.8%	65.3	98.4
**12** T-MSA-Tyr_2_ (623.64)	C (reflux, 22 h)	OH + AA 12.8%; OH + OH 2.3%	31.3	97.0
**13** T-MSA-Trp_2_ (669.71)	A (reflux, 24 h)	OH + OH 2.8%	73.8	97.0
**14** T-MSA-Ser_2_ (471.45)	B (reflux, 7 h)	OH + AA 1%; OH + OH 1%	72.8	98.5
**15** T-MSA-Thr_2_ (499.50)	B (reflux, 7 h)	OH + AA 5.4%	59.8	98.9
**16** T-MSA-Asn_2_ (525.50)	B (reflux, 7 h)	OH + AA 1.7%; OH + OH 1%	76.5	98.1
**17** T-MSA-Gln_2_ (553.55)	B (reflux, 8 h)	OH + AA 31.1%; OH + OH 1.8%	31.6	97.9
**18** T-ESA-Ala_2_ (453.47)	A (reflux, 24 h)	OH + AA 2.7%	82.2	98.5
**19** T-ESA-Tyr_2_ (637.66)	C (reflux, 22 h)	OH + AA 3.3%; OH + OH 1%	20.6	97.8
**20** T-ESA-Trp_2_ (683.74)	A (reflux, 24 h)	OH + OH 1%	76.9	98.4
**21** T-ESA-Ser_2_ (485.47)	B (reflux, 7 h)	OH + AA 1.3%	72.9	97.5
**22** T-ESA-Thr_2_ (513.53)	B (reflux, 7 h)	OH + AA 5.1%	60.5	97.7
**23** T-ESA-Asn_2_ (539.52)	B (reflux, 7 h)	OH + AA 5.1%; OH + OH 7.1%	63.2	98.0
**24** T-ESA-Gln_2_ (567.58)	B (reflux, 8 h)	OH + AA 43.2%; OH + OH 1.2%	29.8	97.1

T-SA = 1,3,5-triazinyl-aminobenzenesulfonamide; T-MSA = 1,3,5-triazinyl-aminomethylbenzenesulfonamide; T-ESA = 1,3,5-triazinyl-aminoethylbenzenesulfonamide; A = water/Na_2_CO_3_; B = water/NaHCO_3_; C = 1,4-dioxane/TEA; main impurities refer to related derivatives, in which chlorines of precursors **1**–**3** are substituted by stated group (hydroxyl OH/amino acid moiety AA).

**Table 2:** Inhibition data for the derivatives **4**–**24** against hCA I, II, IV, IX, XII and their selectivity ratios for tumor-associated isozymes.

			K_i_ (nM)			Selectivity	
Product	hCA I	hCA II	hCA IV	hCA IX	hCA XII	II/IX	II/XII
**4** T-SA-Ala_2_	5545	782	88.1	515	8.88	1.52	88.10
**5** T-SA-Tyr_2_	98.3	3.61	74.6	57.8	9.41	0.06	0.40
**6** T-SA-Trp_2_	48.3	1.61	59.3	45.2	8.21	0.04	0.20
**7** T-SA-Ser_2_	6562	523	8698	465	71.9	1.12	7.30
**8** T-SA-Thr_2_	7273	904	4907	867	34.6	1.04	26.10
**9** T-SA-Asn_2_	>10,000	656	5328	895	38.4	0.73	17.10
**10** T-SA-Gln_2_	9200	775	8411	625	68.3	1.24	11.30
**11** T-MSA-Ala_2_	636	182	470	63.5	9.3	2.87	19.60
**12** T-MSA-Tyr_2_	75.7	53.1	244	87.3	8.7	0.61	6.10
**13** T-MSA-Trp_2_	30.5	6.22	93.6	66.4	8.6	0.09	0.70
**14** T-MSA-Ser_2_	653	252	6098	263	81.5	0.96	3.10
**15** T-MSA-Thr_2_	684	76.6	>10,000	365	44.9	0.21	1.70
**16** T-MSA-Asn_2_	834	251	>10,000	95.3	44.0	2.63	5.70
**17** T-MSA-Gln_2_	695	239	>10,000	458	34.3	0.52	7.00
**18** T-ESA-Ala_2_	787	169	3861	78.8	8.9	2.14	19.00
**19** T-ESA-Tyr_2_	60.8	12.8	879	61.1	7.5	0.21	1.70
**20** T-ESA-Trp_2_	41.4	3.54	542	308	8.4	0.01	0.40
**21** T-ESA-Ser_2_	819	273	>10,000	85.5	27.1	3.19	10.10
**22** T-ESA-Thr_2_	829	259	>10,000	89.5	62.7	2.89	4.10
**23** T-ESA-Asn_2_	886	136	>10,000	377	53.1	0.36	2.60
**24** T-ESA-Gln_2_	684	191	>10,000	480	57.0	0.40	3.40

(i) measured with standard acetazolamide with K_i_ 250, 12, 74, 25 and 5.7 nM; (ii) mean from 3 different assays (errors were in the range of ± 5–10% of the reported values).

**Table 3:** Summary of optimized chromatographic conditions used for the purification of products **11**–**24** by semi-preparative LC with RP-C18 stationary phase.

Product	c (Mobile Phase A) [mmol/L]	Mobile Phase B	Flow Rate [mL/min]	Injected Volume [μL]
**11** T-MSA-Ala_2_	100	MeOH 10%	20	1000
**12** T-MSA-Tyr_2_	50	MeOH 22.5%	20	2500
**13** T-MSA-Trp_2_	100	MeOH 37%	22.5	2000
**14** T-MSA-Ser_2_	50	MeOH 5%	25	2000
**15** T-MSA-Thr_2_	100	MeOH 7.5%	25	1000
**16** T-MSA-Asn_2_	100	MeOH 5%	20	2500
**17** T-MSA-Gln_2_	100	MeCN 4%	20	1000
**18** T-ESA-Ala_2_	100	MeOH 12.5%	20	1000
**19** T-ESA-Tyr_2_	50	MeOH 25%	20	2500
**20** T-ESA-Trp_2_	100	MeOH 40%	20	2500
**21** T-ESA-Ser_2_	50	MeOH 5%	25	2500
**22** T-ESA-Thr_2_	100	MeOH 12.5%	15	1000
**23** T-ESA-Asn_2_	50	MeOH 5%	25	2500
**24** T-ESA-Gln_2_	50	MeCN 5%	20	2000

Mobile phase A ammonium bicarbonate, mobile phase B-MeOH = methanol/MeCN = acetonitrile.

## Data Availability

Data analyzed during the current study are available in this article (or Supplementary material) or are available from the corresponding author on reasonable request.

## Supplementary Materials

The following are available online at https://www.mdpi.com/article/10.3390/ijms222011283/s1.

## Abbreviations

AA	amino acid
DAD	diode-array detector
hCA	human carbonic anhydrase
hCAi	human carbonic anhydrase inhibitors
HILIC	hydrophilic interactions liquid chromatography
HPLC	high performance liquid chromatography
IR	infrared (spectroscopy)
MP	mobile phase
MS	mass spectrometry
NMR	nuclear magnetic resonance
Q-TOF	quadrupole–time-of-flight
RP	reverse phase
TEA	triethylamine
T-SA	1,3,5-triazinyl-aminobenzenesulfonamide derivative
T-MSA	1,3,5-triazinyl-aminomethylbenzenesulfonamide derivative
T-ESA	1,3,5-triazinyl-aminoethylbenzenesulfonamide derivative
