# Supplementary material for: Synthesis and Inhibition Activity Study of Triazinyl-Substituted Amino(alkyl)-benzenesulfonamide Conjugates with Polar and Hydrophobic Amino Acids as Inhibitors of Human Carbonic Anhydrases I, II, IV, IX, and XII
Source: Int J Mol Sci. 2021 Oct 19;22(20):11283. doi: 10.3390/ijms222011283 (PMC8537140; doi:10.3390/ijms222011283)
Supplement: Supplementary file 1 [file ijms-22-11283-s001.zip › ijms-1426343-supplementary.pdf]

Supplementary Material for:

Synthesis and Inhibition Activity Study of Triazinyl-Substituted Amino(alkyl)-Benzenesulfonamide Conjugates with Polar and Hydrophobic Amino Acids as Inhibitors of Human Carbonic Anhydrases I, II, IV, IX, and XII

Mária Bodnár Mikulová [1], Dáša Kružlicová [1], Daniel Pecher [1,2], Andrea Petreni [3], Claudiu T. Supuran [3] and Peter Mikuš [1,2,*]

[1] Department of Pharmaceutical Analysis and Nuclear Pharmacy, Faculty of Pharmacy, Comenius University in Bratislava, Odbojárov 10, 832 32 Bratislava, Slovakia; mikulova43@uniba.sk (M.B.M.); pecher1@uniba.sk (D.P.); kruzlicova@fpharm.uniba.sk (D.K.)

[2] Toxicological and Antidoping Center, Faculty of Pharmacy, Comenius University in Bratislava, Odbojárov 10, 832 32 Bratislava, Slovakia

[3] Neurofarba Department, Section of Pharmaceutical and Nutriceutical Sciences, University of Florence, 50139 Florence, Italy; andrea.petreni@unifi.it (A.P.); claudiu.supuran@unifi.it (C.T.S.).

* Correspondence: mikus@fpharm.uniba.sk (P.M.); Tel.: +421-250117243; ORCID 0000-0001-5755-9970

S1. 2,2′-[(6-({4-sulfamoylbenzyl}amino)-1,3,5-triazine-2,4-diyl)diimino]dipropanoic acid **11** and its IR (a), ^{1}H (b), ^{13}C (c) NMR and LC-DAD/MS (d) spectra

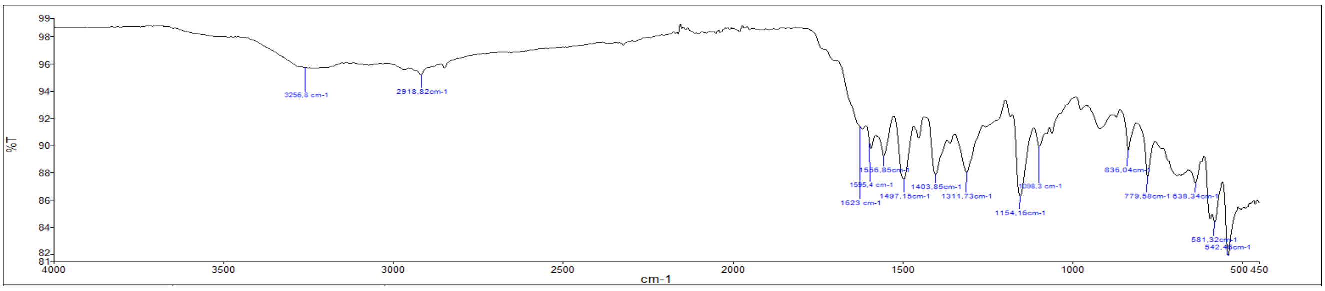

Figure S1a. IR spectra of product **11.**

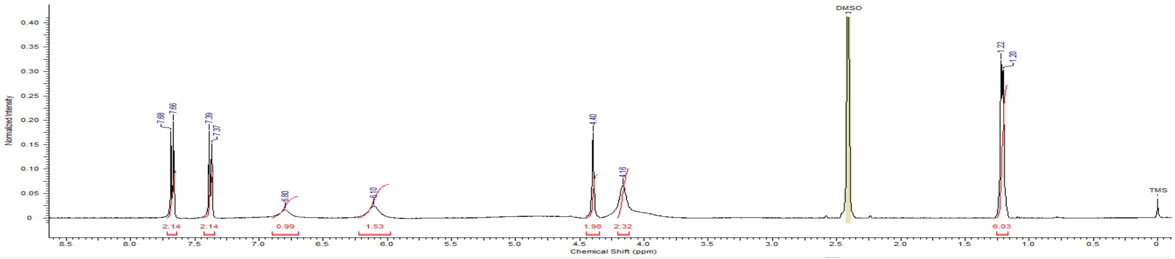

Figure S1b. ^{1}H NMR spectra of product **11.**

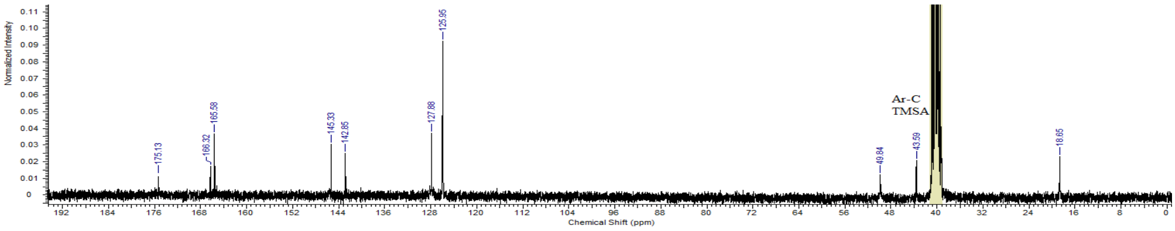

Figure S1c. ^{13}C NMR spectra of product **11.**

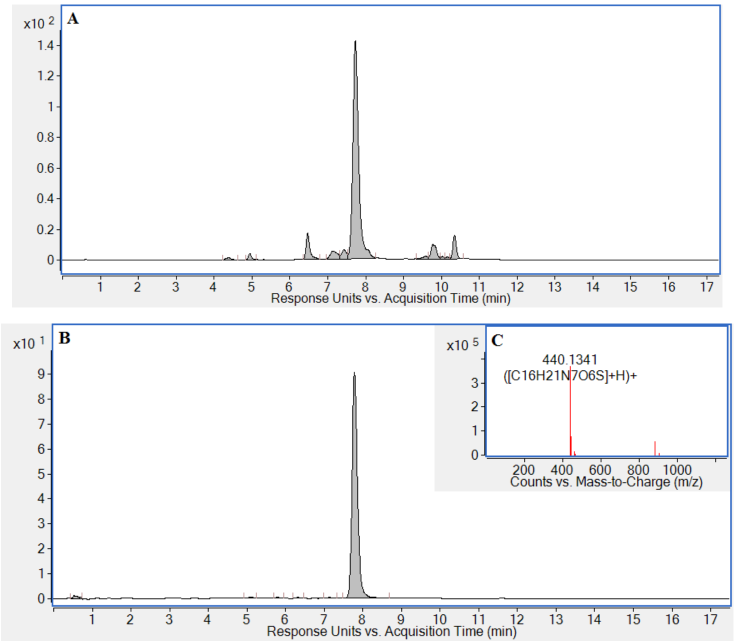

Figure S1d. HILIC-DAD-QTOF analysis of product **11**. Purity profile (HILIC-DAD - 254 nm) before (panel A) and after (panel B) semi-preparative chromatography. Panel C – MS spectrum of the desired compound (the major peak from UV chromatogram).

S2. 2,2′-[(6-({4-sulfamoylbenzyl}amino)-1,3,5-triazine-2,4-diyl)diimino]bis(3-(4-hydroxyphenyl)propanoic acid) **12** and its IR (a), ^{1}H (b), ^{13}C (c) NMR and DAD/MS (d) spectra

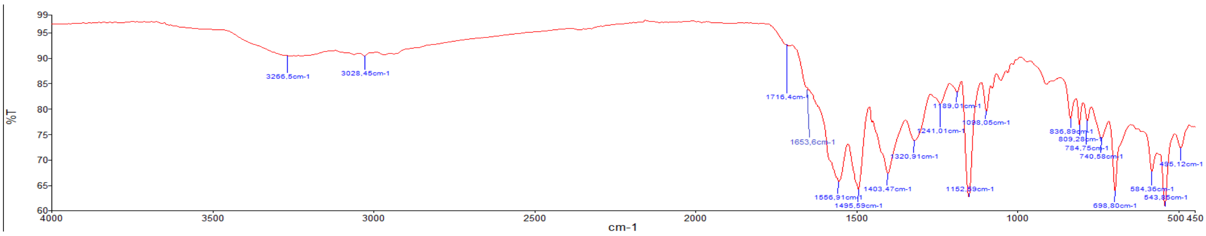

Figure S2a. IR spectra of product **12.**

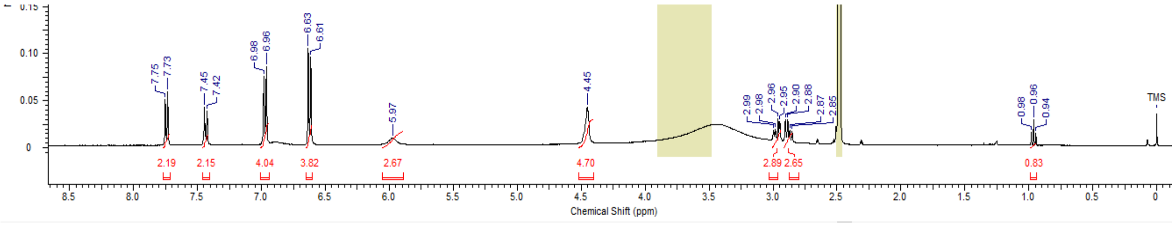

Figure S2b. ^{1}H NMR spectra of product **12.**

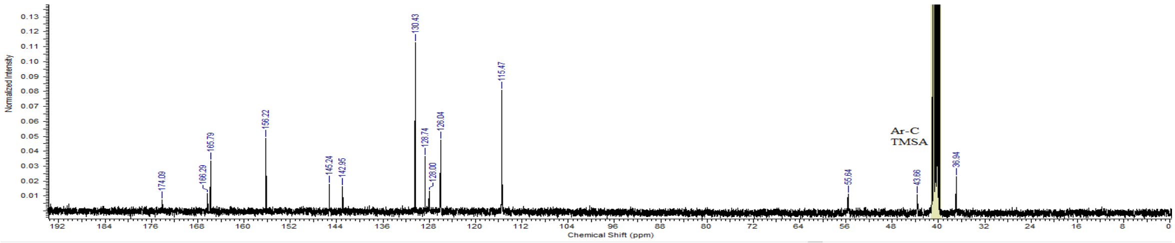

Figure S2c. ^{13}C NMR spectra of product **12.**

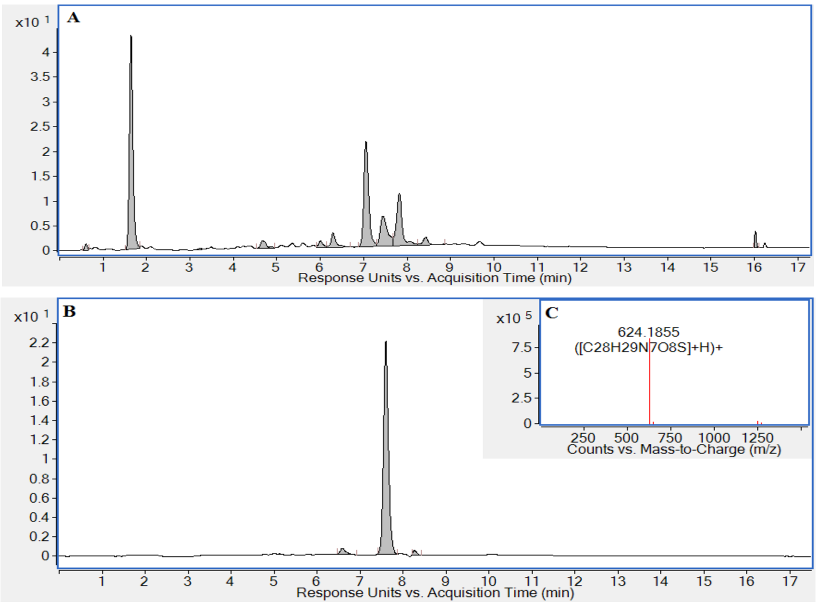

Figure S2d. HILIC-DAD-QTOF analysis of product **12.** Purity profile (HILIC-DAD - 254 nm) before (panel A) and after (panel B) semi-preparative chromatography. Panel C – MS spectrum of the desired compound (the major peak from UV chromatogram).

S3. 2,2′-[(6-({4-sulfamoylbenzyl}amino)-1,3,5-triazine-2,4-diyl)diimino]bis(3-(*1H*-indol-3-yl)propanoic acid) **13** and its IR (a), ¹H (b), ¹³C (c) NMR and DAD/MS (d) spectra

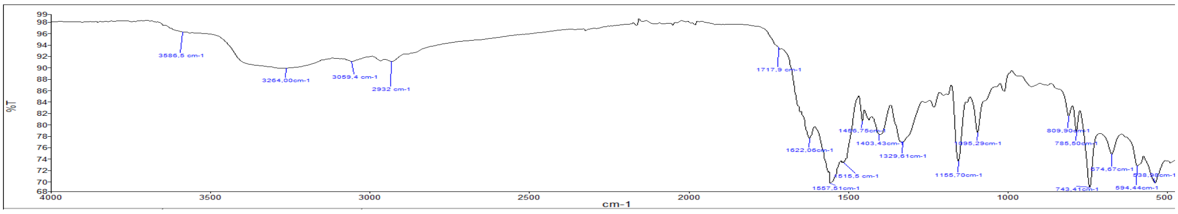

Figure S3a. IR spectra of product **13.**

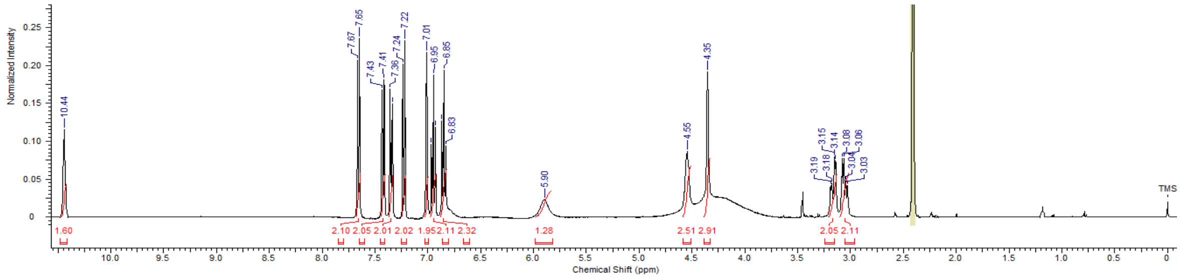

Figure S3b. ¹H NMR spectra of product **13.**

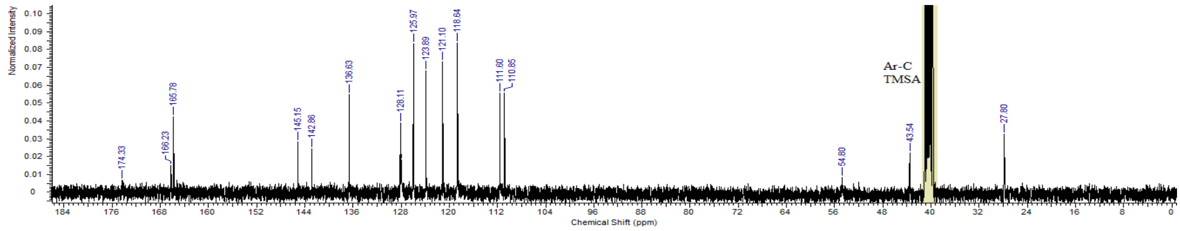

Figure S3c. ¹³C NMR spectra of product **13.**

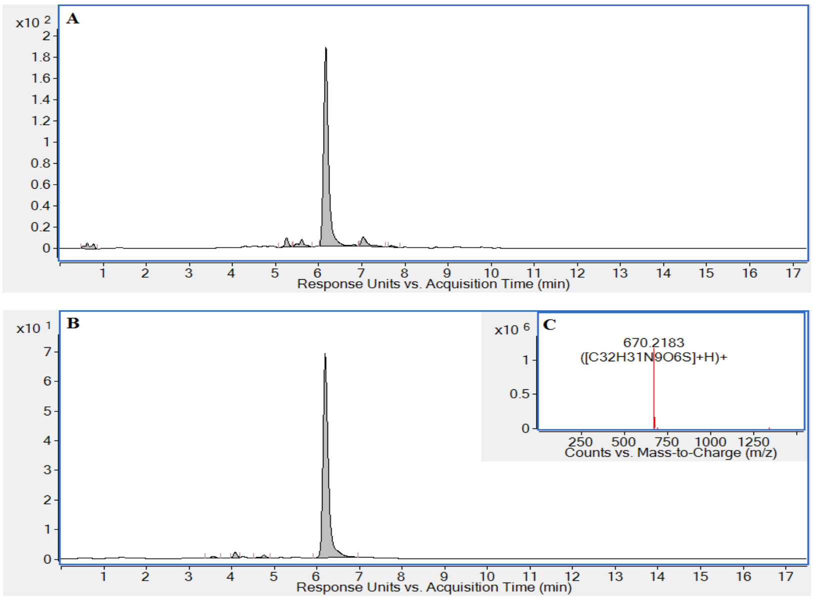

Figure S3d. HILIC-DAD-QTOF analysis of product **13**. Purity profile (HILIC-DAD - 254 nm) before (panel A) and after (panel B) semi-preparative chromatography. Panel C – MS spectrum of the desired compound (the major peak from UV chromatogram).

S4. 2,2′-[(6-({4-sulfamoylbenzyl}amino)-1,3,5-triazine-2,4-diyl)diimino]bis(3-hydroxypropanoic acid) **14** and its IR (a), ^{1}H (b), ^{13}C (c) NMR and DAD/MS (d) spectra

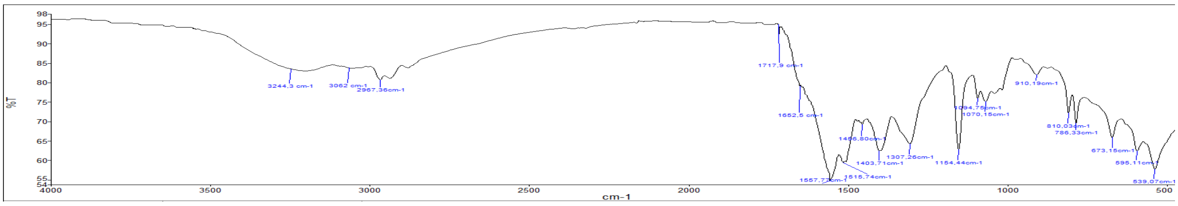

Figure S4a. IR spectra of product **14.**

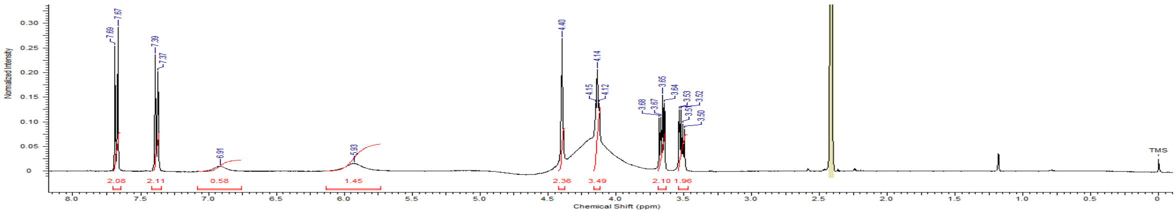

Figure S4b. ^{1}H NMR spectra of product **14.**

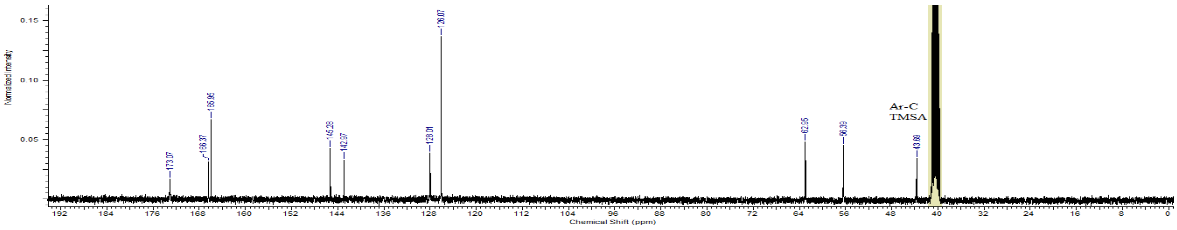

Figure S4c. ^{13}C NMR spectra of product **14.**

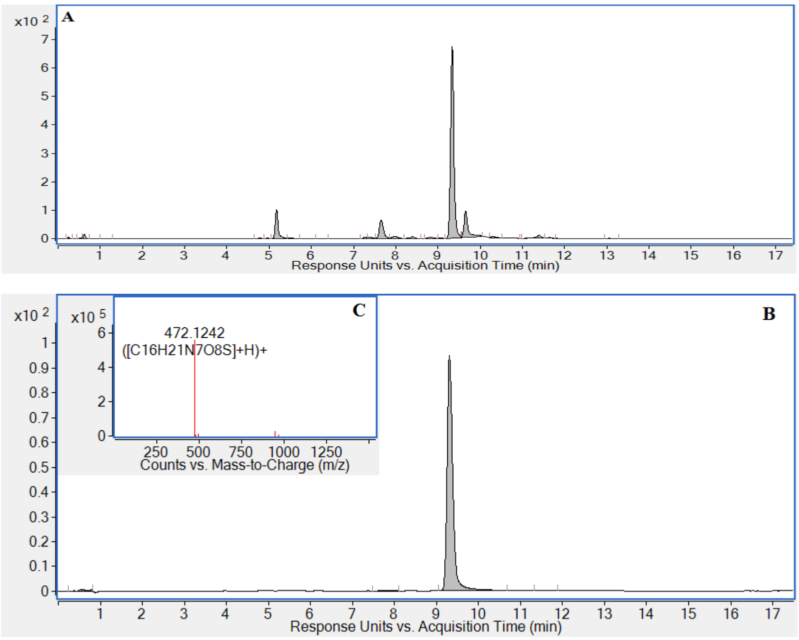

Figure S4d. HILIC-DAD-QTOF analysis of product **14**. Purity profile (HILIC-DAD - 254 nm) before (panel A) and after (panel B) semi-preparative chromatography. Panel C – MS spectrum of the desired compound (the major peak from UV chromatogram).

__S5.__ 2,2′-[(6-({4-sulfamoylbenzyl}amino)-1,3,5-triazine-2,4-diyl)diimino]bis(3-hydroxybutanoic acid) **15** and its IR (a), ^{1}H (b), ^{13}C (c) NMR and DAD/MS (d) spectra.

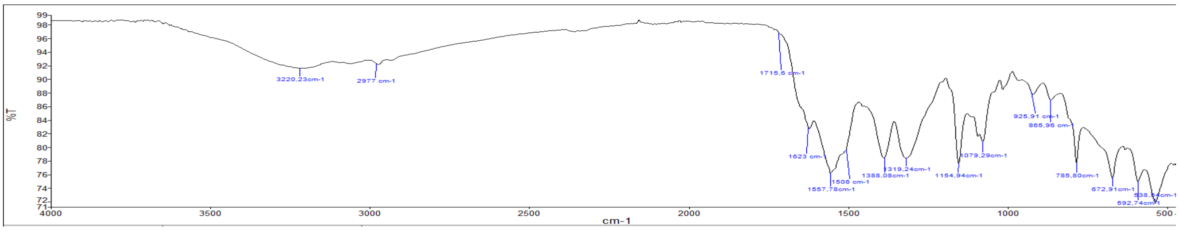

Figure S5a. IR spectra of product **15.**

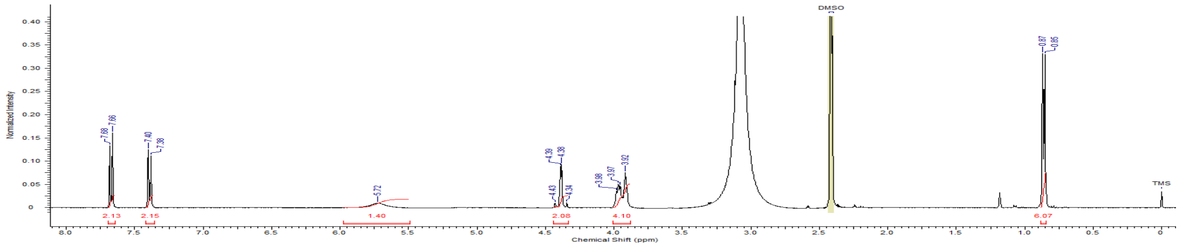

Figure S5b. ^{1}H NMR spectra of product **15.**

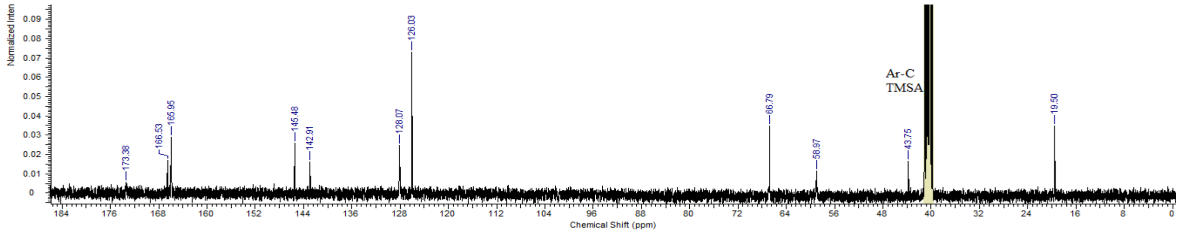

Figure S5c. ^{13}C NMR spectra of product **15.**

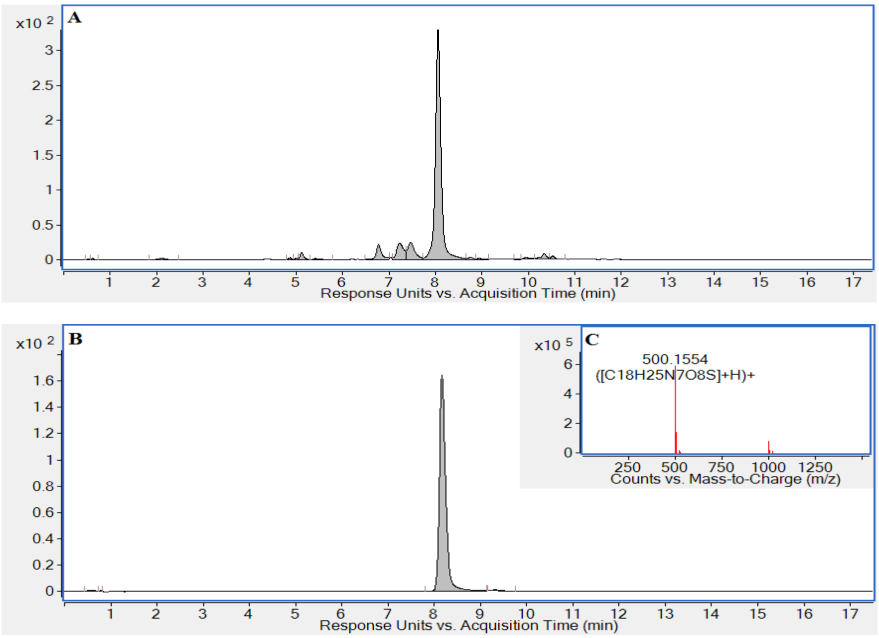

Figure S5d. HILIC-DAD-QTOF analysis of product **15**. Purity profile (HILIC-DAD - 254 nm) before (panel A) and after (panel B) semi-preparative chromatography. Panel C – MS spectrum of the desired compound (the major peak from UV chromatogram).

S6. 2,2′-[(6-({4-sulfamoylbenzyl}amino)-1,3,5-triazine-2,4-diyl)diimino]bis(3-carbamoylpropanoic acid) **16** and its IR (a), ^{1}H (b), ^{13}C (c) NMR and DAD/MS (d) spectra.

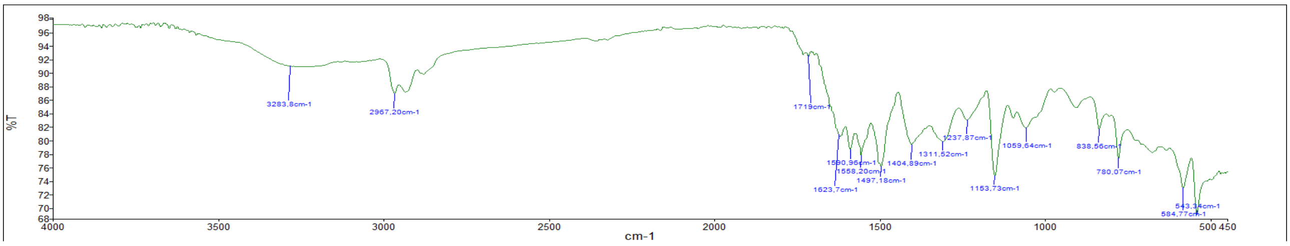

Figure S6a. IR spectra of product **16.**

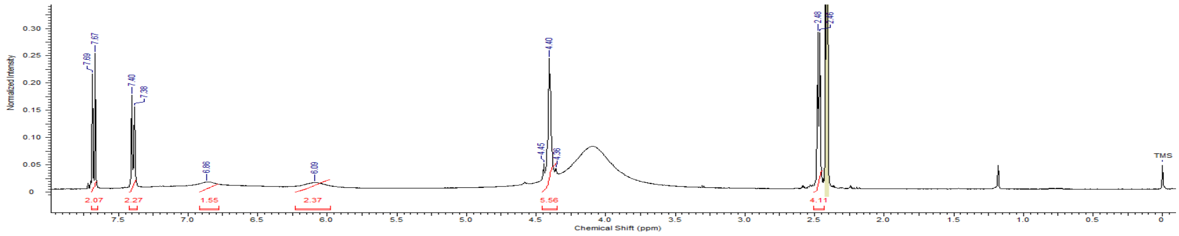

Figure S6b. ^{1}H NMR spectra of product **16.**

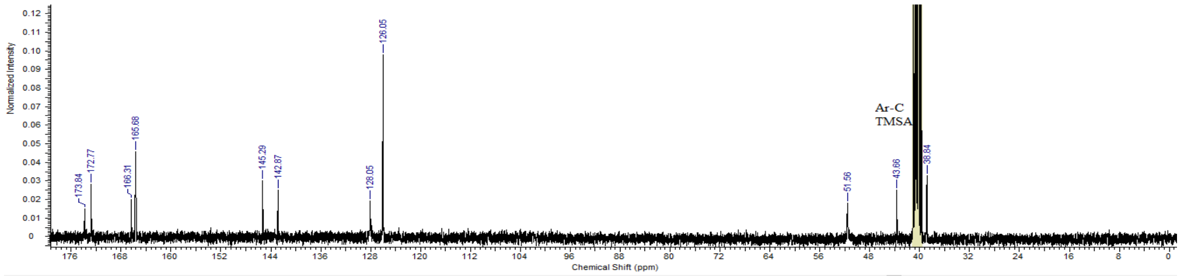

Figure S6c. ^{13}C NMR spectra of product **16.**

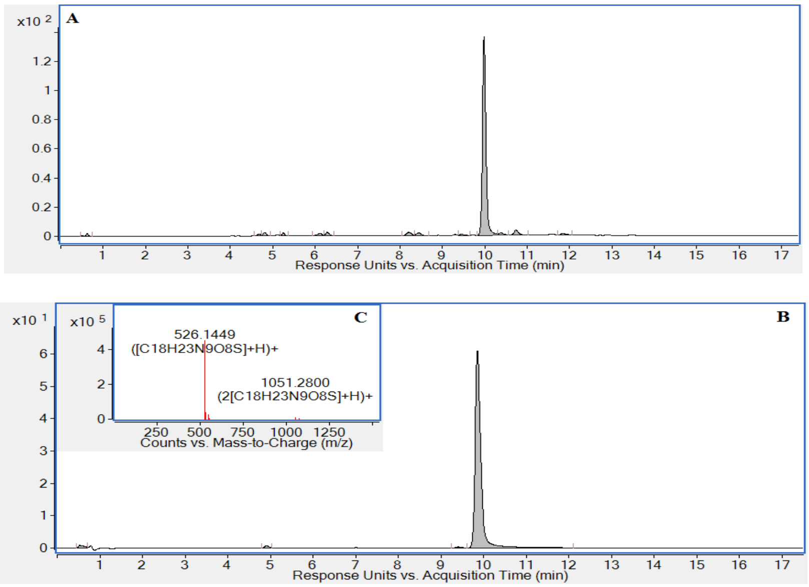

Figure S6d. HILIC-DAD-QTOF analysis of product **16**. Purity profile (HILIC-DAD - 254 nm) before (panel A) and after (panel B) semi-preparative chromatography. Panel C – MS spectrum of the desired compound (the major peak from UV chromatogram).

S7. 2,2′-[(6-({4-sulfamoylbenzyl}amino)-1,3,5-triazine-2,4-diyl)diimino]bis(4-carbamoylbutanoic acid) **17** and its IR (a), ^{1}H (b), ^{13}C (c) NMR and DAD/MS (d) spectra

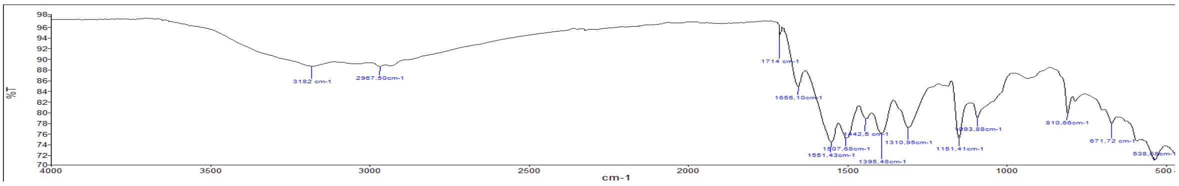

Figure S7a. IR spectra of product **17.**

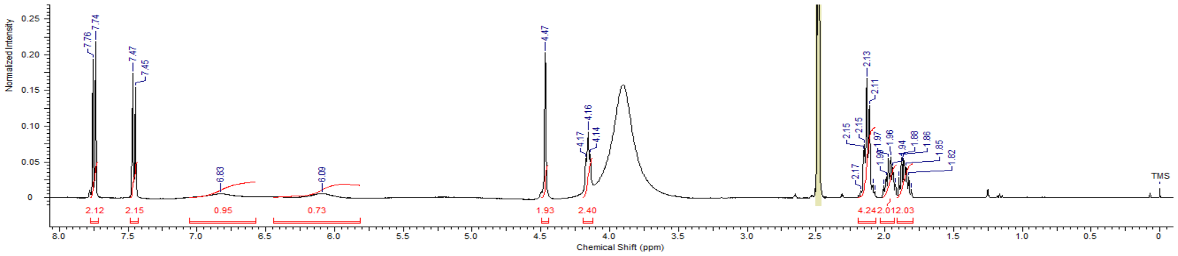

Figure S7b. ^{1}H NMR spectra of product **17.**

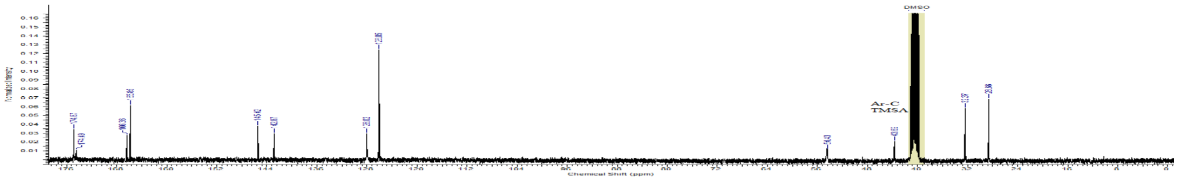

Figure S7c. ^{13}C NMR spectra of product **17.**

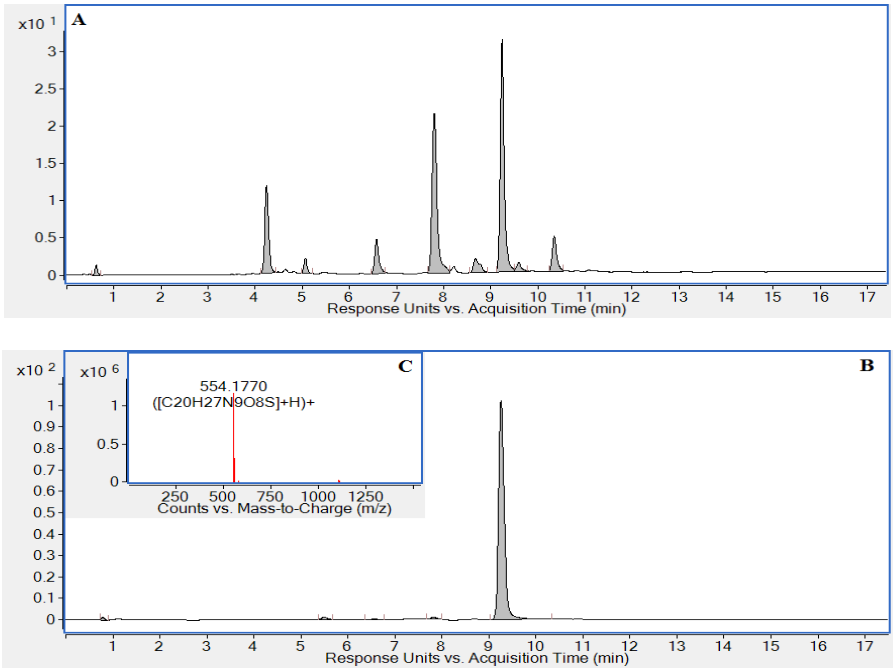

Figure S7d. HILIC-DAD-QTOF analysis of product **17**. Purity profile (HILIC-DAD - 254 nm) before (panel A) and after (panel B) semi-preparative chromatography. Panel C – MS spectrum of the desired compound (the major peak from UV chromatogram).

__S8__. 2,2′-[(6-({2-(4-sulfamoylphenyl)ethyl}amino)-1,3,5-triazine-2,4-diyl)diimino]dipropanoic acid **18** and its IR (a), ^{1}H (b), ^{13}C (c) NMR and DAD/MS (d) spectra

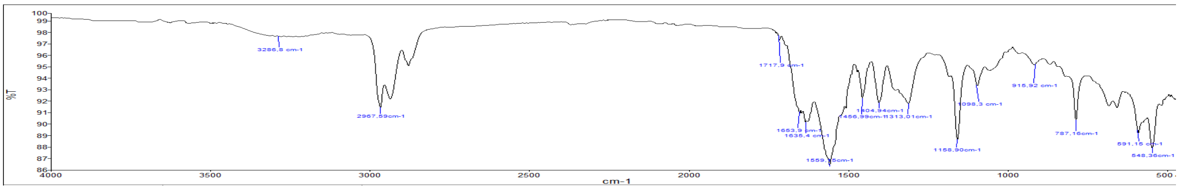

Figure S8a. IR spectra of product **18.**

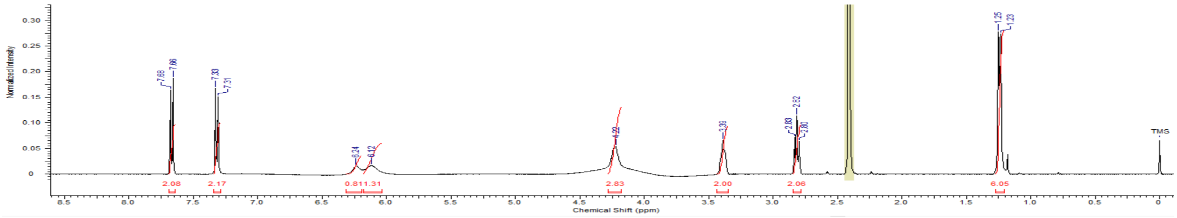

Figure S8b. ^{1}H NMR spectra of product **18.**

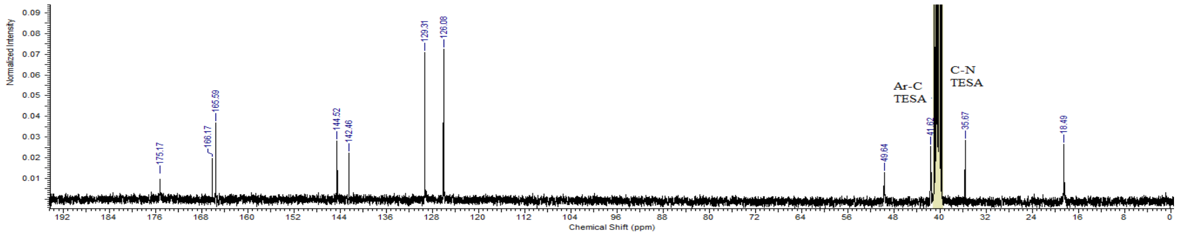

Figure S8c. ^{13}C NMR spectra of product **18.**

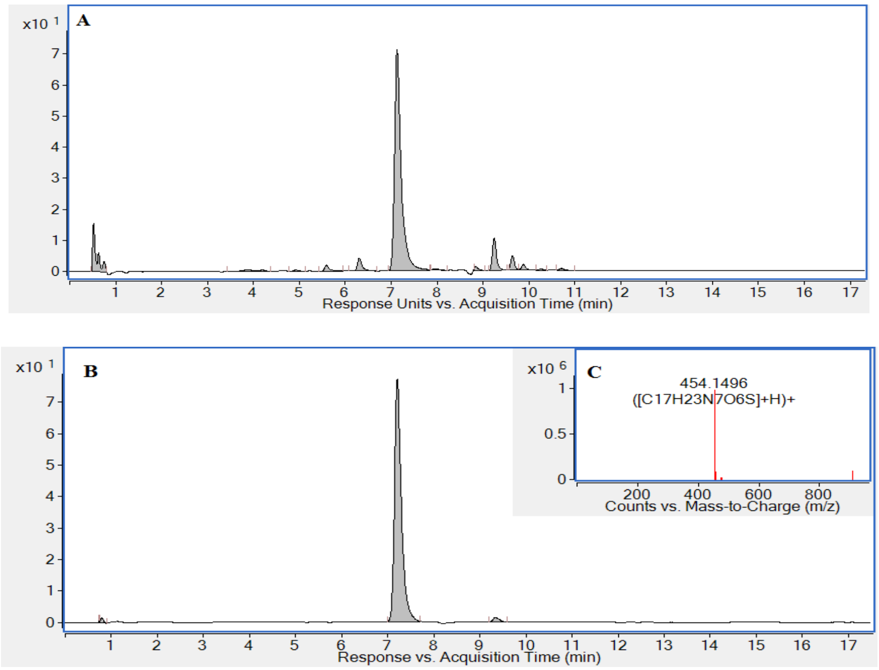

Figure S8d. HILIC-DAD-QTOF analysis of product **18.** Purity profile (HILIC-DAD - 254 nm) before (panel A) and after (panel B) semi-preparative chromatography. Panel C – MS spectrum of the desired compound (the major peak from UV chromatogram).

S9. 2,2′-[(6-({2-(4-sulfamoylphenyl)ethyl}amino)-1,3,5-triazine-2,4-diyl)diimino]bis(3-(4-hydroxyphenyl)propanoic acid) **19** and its IR (a), ^{1}H (b), ^{13}C (c) NMR and DAD/MS (d) spectra.

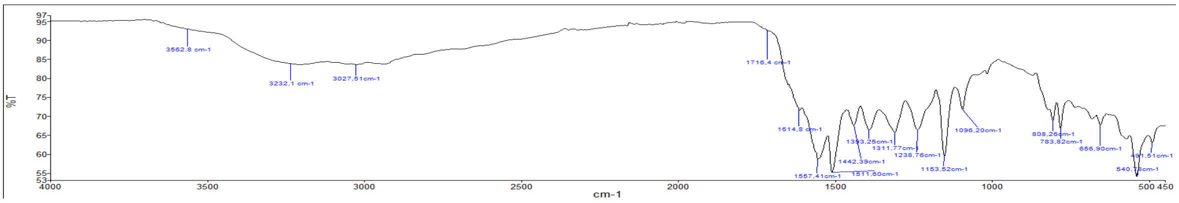

Figure S9a. IR spectra of product **19.**

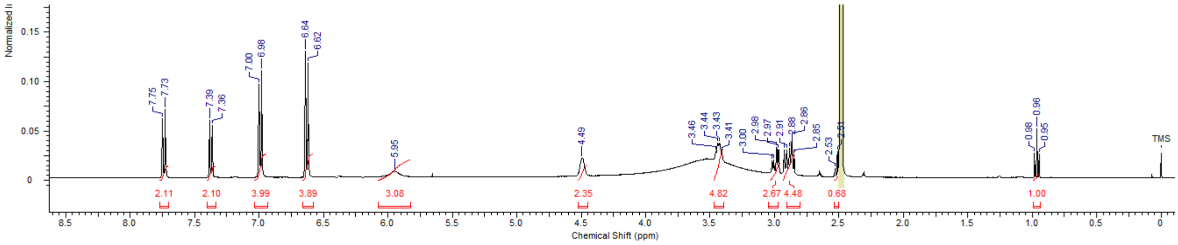

Figure S9b. ^{1}H NMR spectra of product **19.**

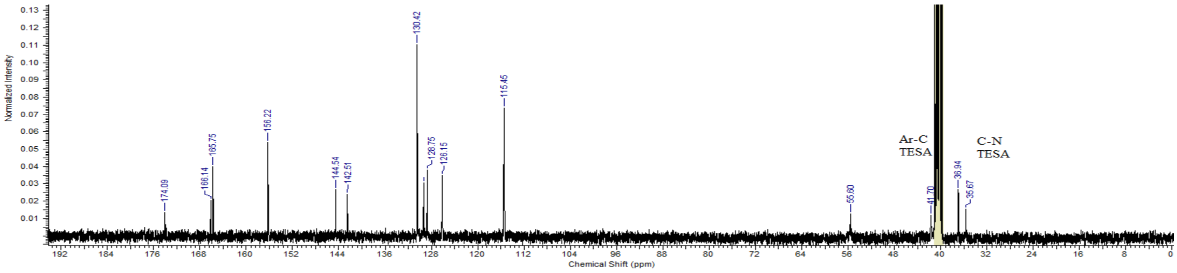

Figure S9c. ^{13}C NMR spectra of product **19.**

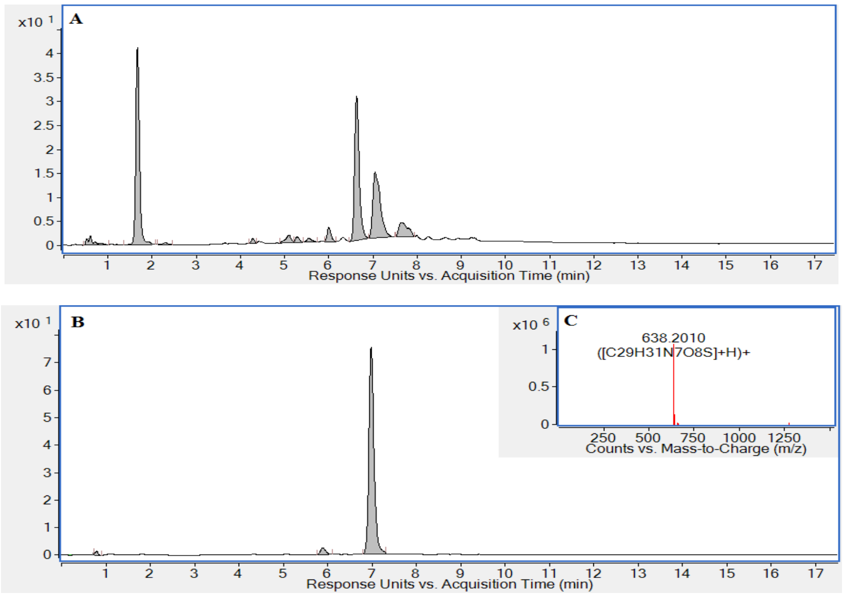

Figure S9d. HILIC-DAD-QTOF analysis of product **19**. Purity profile (HILIC-DAD - 254 nm) before (panel A) and after (panel B) semi-preparative chromatography. Panel C – MS spectrum of the desired compound (the major peak from UV chromatogram).

S10. 2,2′-[(6-({2-(4-sulfamoylphenyl)ethyl}amino)-1,3,5-triazine-2,4-diyl)diimino]bis(3-(*1H*-indol-3-yl)propanoic acid) **20** and its IR (a), ^{1}H (b), ^{13}C (c) NMR and DAD/MS (d) spectra

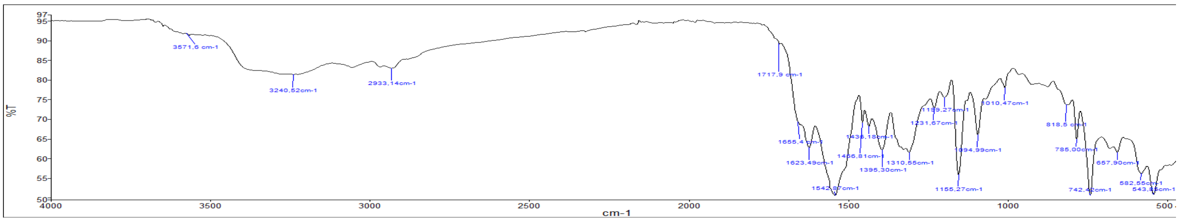

Figure S10a. IR spectra of product **20.**

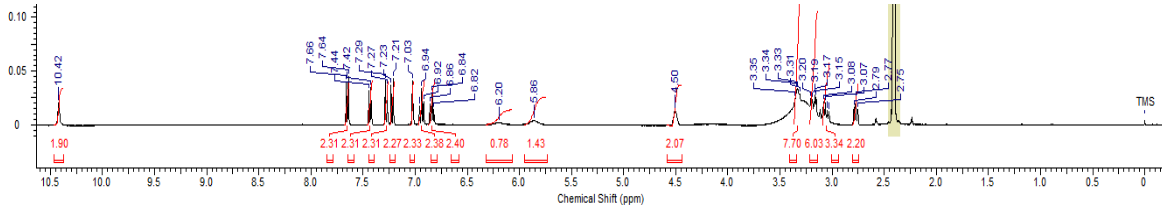

Figure S10b. ^{1}H NMR spectra of product **20.**

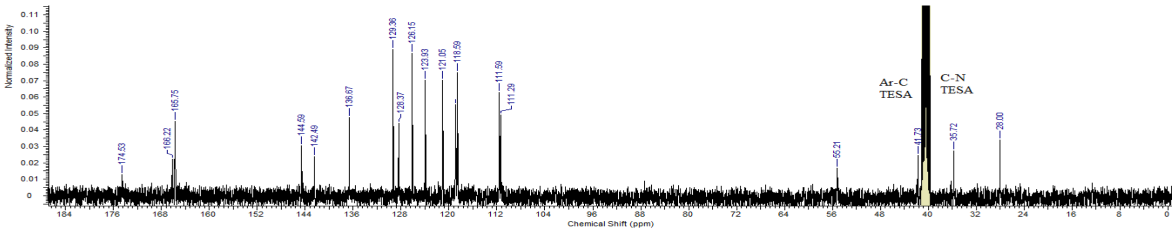

Figure S10c. ^{13}C NMR spectra of product **20.**

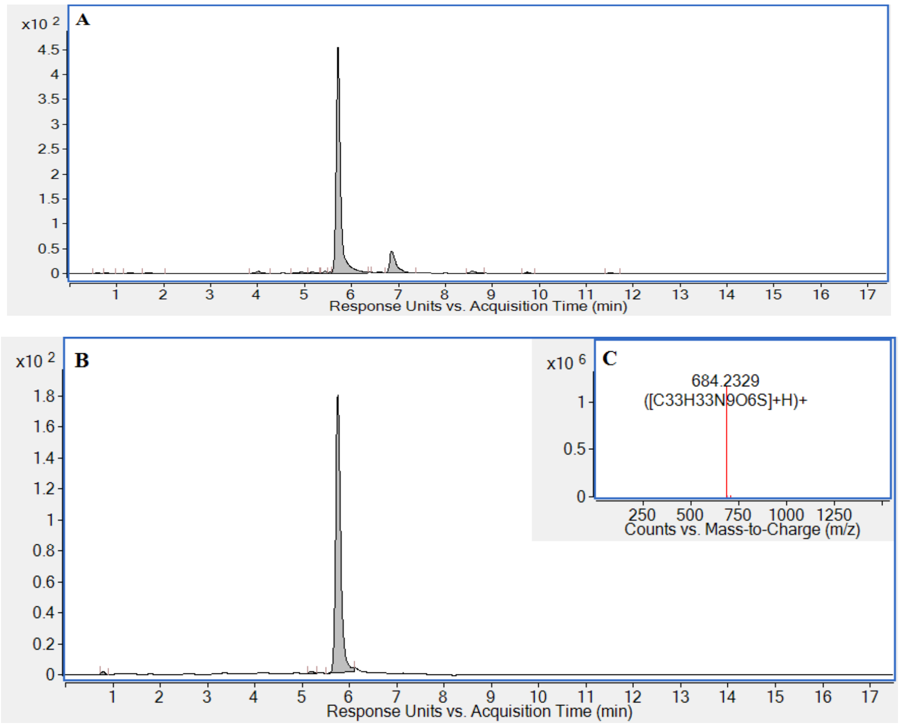

Figure S10d. HILIC-DAD-QTOF analysis of product **20**. Purity profile (HILIC-DAD - 254 nm) before (panel A) and after (panel B) semi-preparative chromatography. Panel C – MS spectrum of the desired compound (the major peak from UV chromatogram).

S11. 2,2′-[(6-({2-(4-sulfamoylphenyl)ethyl}amino)-1,3,5-triazine-2,4-diyl)diimino]bis(3-hydroxypropanoic acid) **21** and its IR (a), ^{1}H (b), ^{13}C (c) NMR and DAD/MS (d) spectra

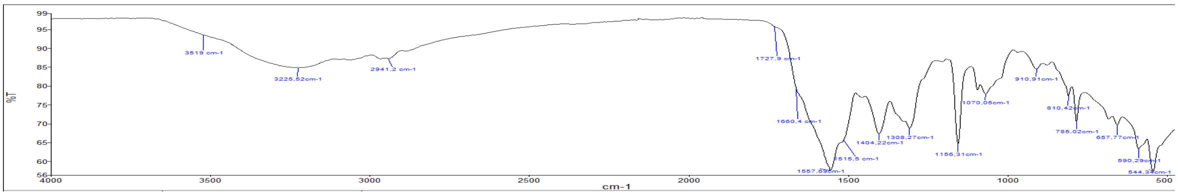

Figure S11a. IR spectra of product **21.**

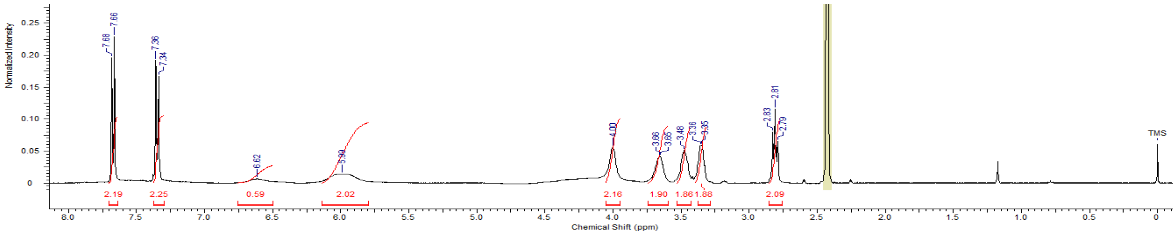

Figure S11b. ^{1}H NMR spectra of product **21.**

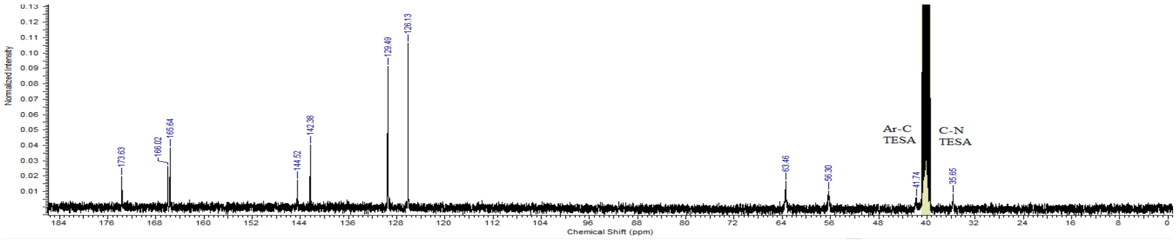

Figure S11c. ^{13}C NMR spectra of product **21.**

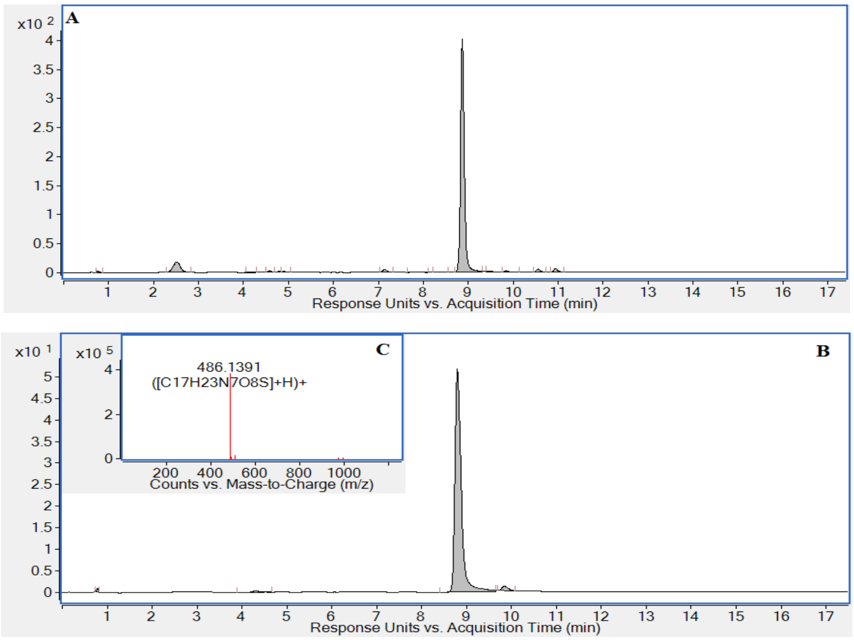

Figure S11d. HILIC-DAD-QTOF analysis of product **21**. Purity profile (HILIC-DAD - 254 nm) before (panel A) and after (panel B) semi-preparative chromatography. Panel C – MS spectrum of the desired compound (the major peak from UV chromatogram).

S12. 2,2′-[(6-({2-(4-sulfamoylphenyl)ethyl}amino)-1,3,5-triazine-2,4-diyl)diimino]bis(3-hydroxybutanoic acid) **22** and its IR (a), ^{1}H (b), ^{13}C (c) NMR and DAD/MS (d) spectra

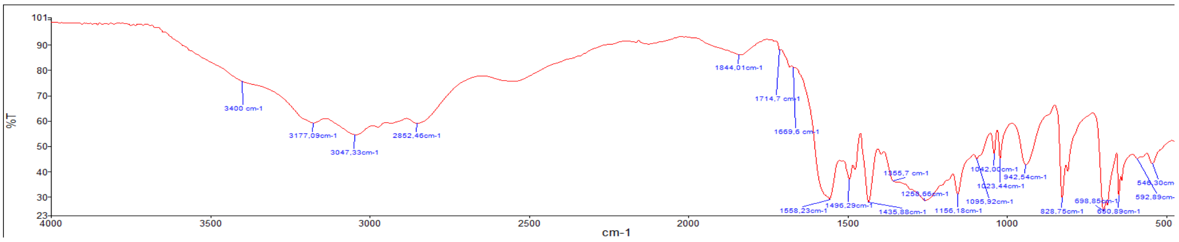

Figure S12a. IR spectra of product **22.**

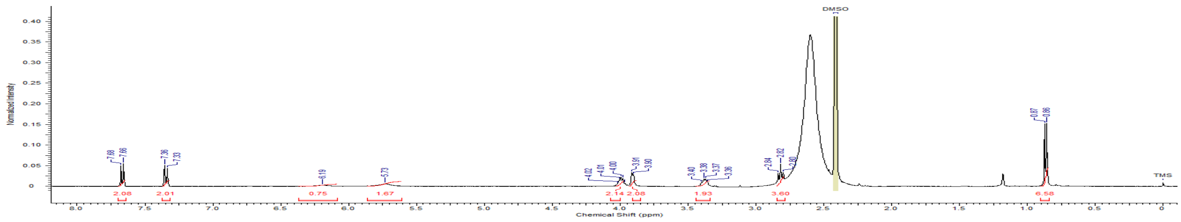

Figure S12b. ^{1}H NMR spectra of product **22.**

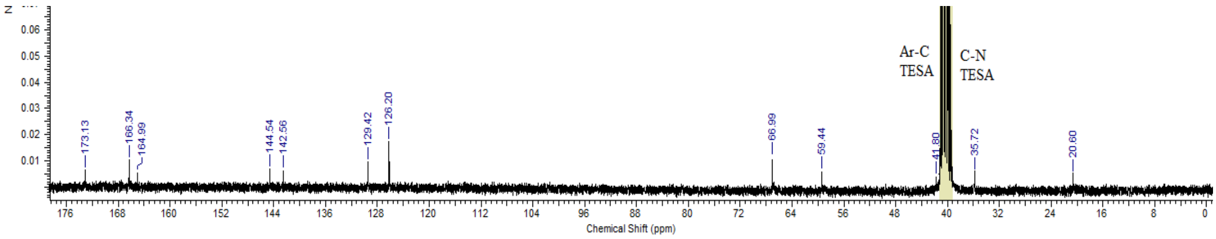

Figure S12c. ^{13}C NMR spectra of product **22.**

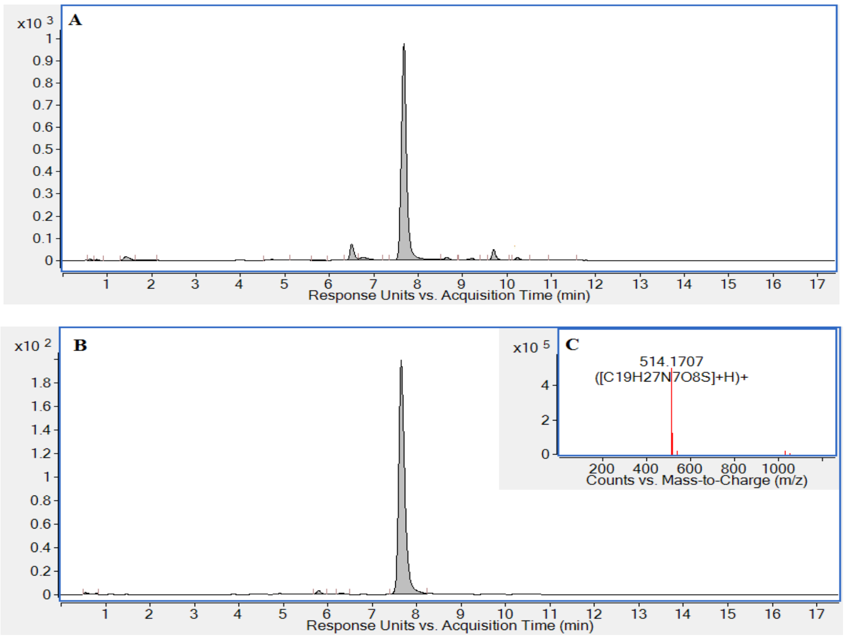

Figure S12d. HILIC-DAD-QTOF analysis of product **22**. Purity profile (HILIC-DAD - 254 nm) before (panel A) and after (panel B) semi-preparative chromatography. Panel C – MS spectrum of the desired compound (the major peak from UV chromatogram).

S13. 2,2′-[(6-({2-(4-sulfamoylphenyl)ethyl}amino)-1,3,5-triazine-2,4-diyl)diimino]bis(3-carbamoylpropanoic acid) **23** and its IR (a), ^{1}H (b), ^{13}C (c) NMR and DAD/MS (d) spectra

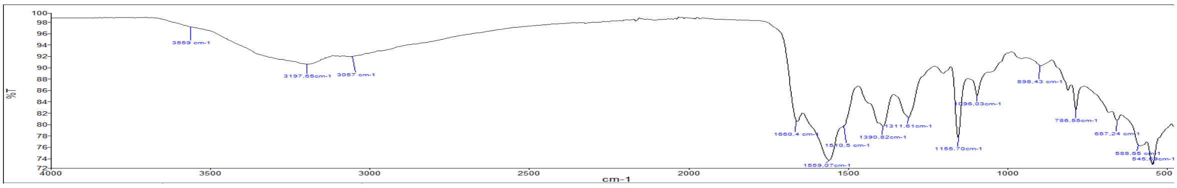

Figure S13a. IR spectra of product **23.**

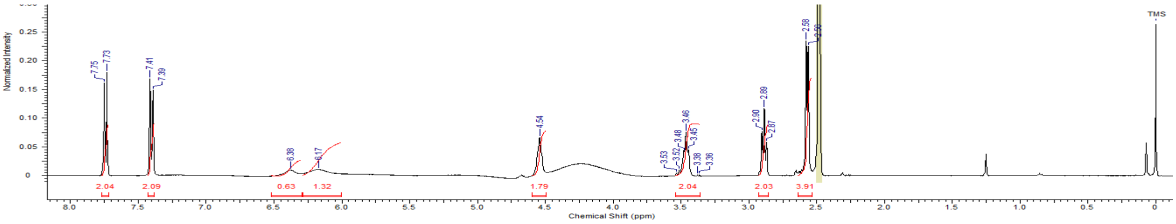

Figure S13b. ^{1}H NMR spectra of product **23.**

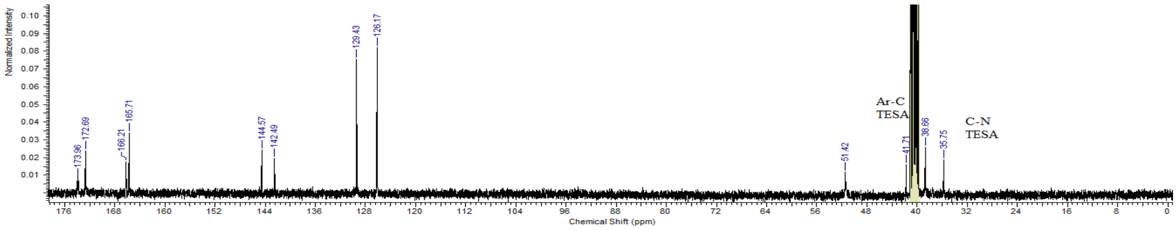

Figure S13c. ^{13}C NMR spectra of product **23.**

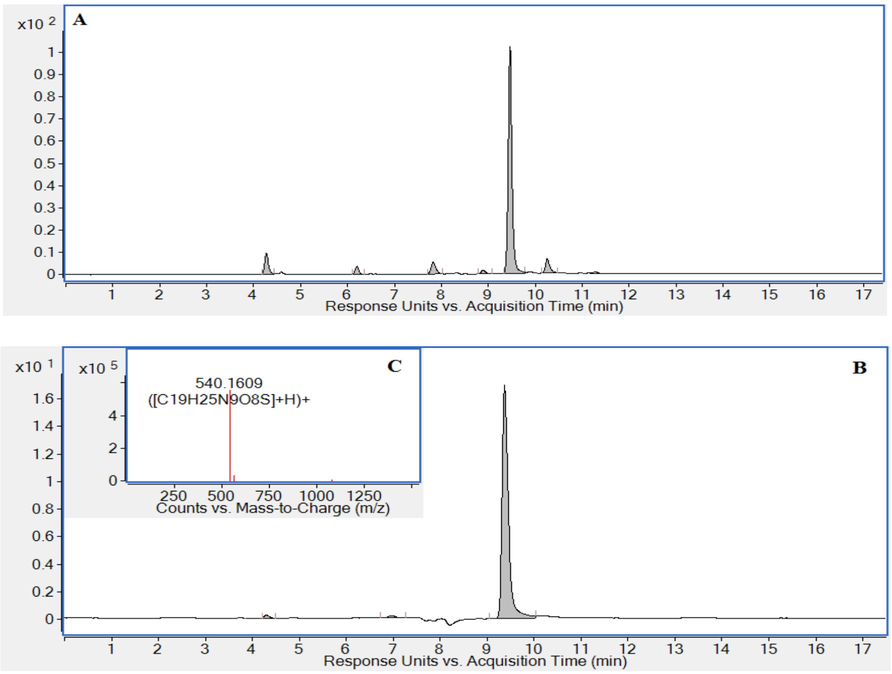

Figure S13d. HILIC-DAD-QTOF analysis of product **23**. Purity profile (HILIC-DAD - 254 nm) before (panel A) and after (panel B) semi-preparative chromatography. Panel C – MS spectrum of the desired compound (the major peak from UV chromatogram).

S14. 2,2′-[(6-({2-(4-sulfamoylphenyl)ethyl}amino)-1,3,5-triazine-2,4-diyl)diimino]bis(4-carbamoylbutanoic acid) **24** and its IR (a), ^{1}H (b), ^{13}C (c) NMR and DAD/MS (d) spectra

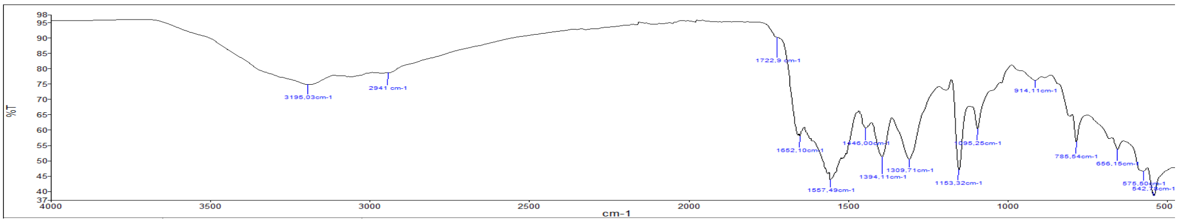

Figure S14a. IR spectra of product **24.**

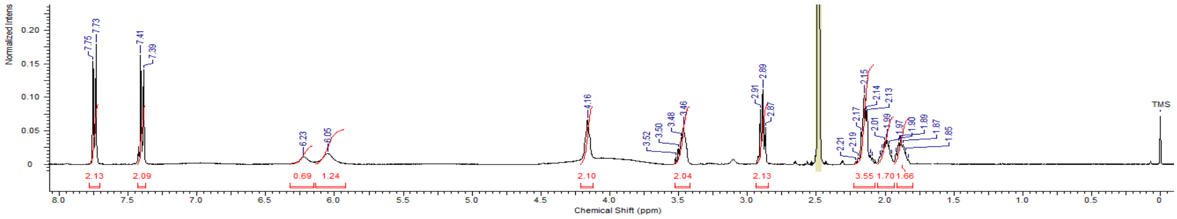

Figure S14b. ^{1}H NMR spectra of product **24.**

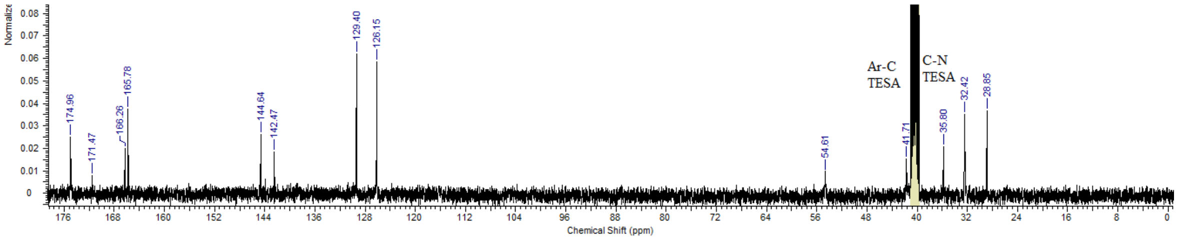

Figure S14c. ^{13}C NMR spectra of product **24.**

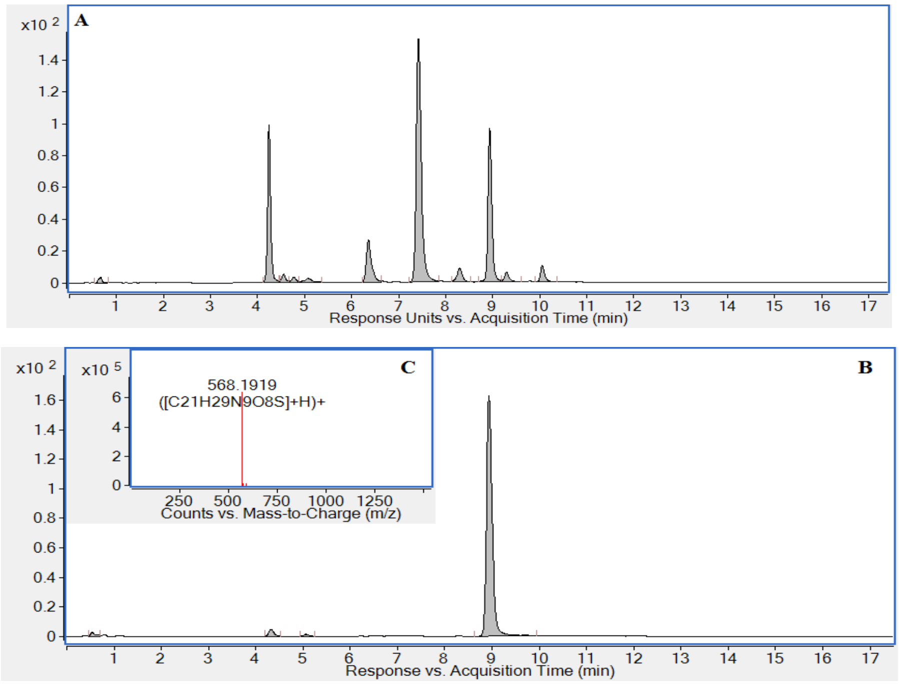

Figure S14d. HILIC-DAD-QTOF analysis of product **24**. Purity profile (HILIC-DAD - 254 nm) before (panel A) and after (panel B) semi-preparative chromatography. Panel C – MS spectrum of the desired compound (the major peak from UV chromatogram).
